# Salinity stress tolerance in *Vigna* species: insights into adaptive responses and innovative approaches for sustainable agriculture

**DOI:** 10.3389/fpls.2025.1735056

**Published:** 2026-01-20

**Authors:** Sekar Suriya, Chinnadurai Immanuel Selvaraj

**Affiliations:** 1 Research Scholar, Agricultural Biotechnology, VIT School of Agricultural Innovations and Advanced Learning (VAIAL), Vellore Institute of Technology, Vellore, Tamil Nadu, India; 2 Department of Genetics and Plant Breeding, VIT School of Agricultural Innovations and Advanced Learning (VAIAL), Vellore Institute of Technology, Vellore, Tamil Nadu, India

**Keywords:** food security, oxidative stress, salinity, stress mitigation, tolerance mechanisms, *Vigna* species

## Abstract

Legumes are widely recognised as important protein sources; among them, *Vigna* species are recognised for their substantial protein content, approximately 20–25%, and play an important role in global nutrition. However, yield is severely reduced when groundwater with elevated salinity levels is used for irrigation. Salinity is a significant abiotic stress that substantially decreases agricultural yield, particularly in irrigated and less fertile areas. In *Vigna* species, salt stress causes impaired seed germination, delayed seedling growth, nutrient uptake deficits, inhibition of photosynthetic mechanisms, disruption of plant hormone homeostasis and oxidative stress. The primary cause of these effects is due to the excessive accumulation of Na^+^ and Cl^-^, which leads to ion toxicity and significant yield loss. To minimise these effects, *Vigna* species have certain adaptation strategies such as antioxidant enzyme activity, osmoprotectant accumulation, vacuolar compartmentalisation, ion exclusion, hormonal adjustment, and maintenance of ion homeostasis. Besides inherent tolerance mechanisms, recent approaches such as the exogenous application of nutrients, biostimulants, seed priming, plant growth-promoting rhizobacteria and nanotechnology can improve plant nutrient uptake and stress resilience under salinity. Furthermore, biotechnology interventions such as marker-assisted breeding, transgenic approaches, gene editing, and omics-based strategies have promising potential for developing salt-tolerant *Vigna* genotypes. This review specifically examines the physiological, biochemical, and molecular responses of *Vigna* species to salinity and evaluates various mitigation strategies. By highlighting recent advances and identifying key research gaps, this review provides insights for developing integrated and sustainable approaches to enhance salinity tolerance in *Vigna* species, thereby supporting sustainable agriculture and global food security.

## 1 Introduction

The global population is expected to reach nearly 10 billion by 2050, increasing global food demand by approximately 50% by 2050 (FAO, 2018; Gauchan et al., 2022). To address this growing need, it is necessary to increase the agricultural productivity, especially in cereals and legumes, by at least 87% compared to current crop production levels (Kromdijk and Long, 2016; Nadeem et al., 2019). To meet this rising food demand, promoting underutilised crops, particularly legumes, will substantially decrease food insecurity due to their inherent characteristics, such as the ability to withstand drought, their high nutritional value, and fixing atmospheric nitrogen, which promotes plant growth, yield, improves soil fertility and food production (Ayilara et al., 2022; Olanrewaju et al., 2022). The genus *Vigna* of the Fabaceae (Legume) family comprises more than 100 wild species and is taxonomically classified into five subgenera: Vigna, Ceratotropis, Haydonia, Lasiospron, and Plectrotropis. Within the genus, 10 crop species have been domesticated, all from three subgenera namely, Ceratotropis, Plectrotropis, and Vigna. The most significant taxonomic group in agriculture is the subgenus Ceratotropis, also called the *Asian Vigna*. From this group, seven crops have been domesticated: the adzuki bean [*Vigna angularis* (Willd.) Ohwi and Ohashi], the mung bean [*Vigna radiata* (L.) R. Wilczek], the black gram [*Vigna mungo* (L.) Hepper], the creole bean [*Vigna reflexo*-pilosa Hayata], the rice bean [*Vigna umbellata* (Thunb.) Ohwi and Ohashi], the minni payaru [*Vigna stipulacea* Kuntze], and the moth bean [*Vigna aconitifolia* (Jacq.) Maréchal] (Takahashi et al., 2016; Verma et al., 2022). “Tuber cowpea”, also known as [*Vigna vexillata* (L.) A. Rich.], is a lesser-known but potentially significant food legume that belongs to the subgenus Plectrotropis (Karuniawan et al., 2006). Cowpea [*Vigna unguiculata* (L.) Walp.] and bambara groundnut [*Vigna subterranean* (L.) Verdc.] are the domesticated species from the subgenus *Vigna* (Maréchal, 1978).

Among several legumes, *Vigna* species are mostly grown in tropical and subtropical regions of Asia, Africa, Australia, and the Pacific regions. Globally, about 32 million metric tonnes (MMT) of *Vigna* species are cultivated each year, with Asia and Africa producing more than half of the World’s production (Rawal and Navarro, 2019). At the global level, mung bean and cowpea are the two most important domesticated *Vigna* species in terms of production. In 2020, 15 million hectares of agricultural land produced 8.9 million tonnes (Mt) of cowpeas globally (Verma et al., 2022). Mung bean production has risen by 2.5% every year over the past decade. The reason for this development is that it fits into many crop rotation cycles due to its short crop life span. Consequently, 7.3 million hectares of land produced 5.3 million tonnes of mung beans in 2020 (Nair and Schreinemachers, 2020; Verma et al., 2022).

*Vigna* species like cowpea, urd bean, and mung bean, which are important for global food security because of their high protein content and efficient nitrogen-fixing ability (Dilipan and Nisha, 2024). The protein content of major legume crops varies substantially, ranging from 19–27% in chickpea, 21–31% in lentil, and 26–33% in fava bean, while *Vigna* species such as bambara groundnut (19-23%), urd bean (20-25%) (Kanth et al., 2021), moth bean (23–26%), cowpea (24–28%), and mung bean (15–33%) and fall within the higher end of this range. This comparison highlights the strong nutritional value of *Vigna* species relative to other legumes (Bennetau-Pelissero, 2018; Bhadkaria et al., 2022). These plants are also beneficial for sustainable agriculture because they increase soil fertility and reduce the need for chemical fertilisers. However, while *Vigna* species may survive in adverse conditions, they are more vulnerable to salt stress than cereals (Ali et al., 2023). This vulnerability can interfere with germination, development, nitrogen fixation and reproduction, which can lead to problems like wilting and delayed maturity (Dilipan and Nisha, 2024).

Salinity stress, which reduces yield and impacts food security, is one of the most important abiotic challenges facing agriculture globally (Stavi et al., 2021). According to the Food and Agriculture Organisation (FAO), salinity impacts about 424 million hectares of topsoil and 833 million hectares of subsoil worldwide, representing a substantial portion of global agricultural land (FAO, 2021; Khalifa et al., 2024) and future predictions indicate that more than 50% of agricultural land will be at risk of becoming salt-affected by 2050 (FAO and ITPS, 2015; Hussain et al., 2019; Kumawat et al., 2024; Wang et al., 2024). This problem is most critical in arid and semi-arid areas of the world’s agricultural land. In India, especially in the Indo-Gangetic plains, regions like Gujarat, Tamil Nadu, and Punjab have already seen significant impacts due to saline conditions (Kumawat et al., 2021, 2024).

Saline soils include several dissolved salts, including NaCl, MgSO_4_, Na_2_SO_4_, KCl, MgCl_2_, CaSO_4_, and Na_2_CO_3_, which may lead to salt stress. NaCl is the most prevalent reason for salinity and has been the focus of many studies (Al-Saady et al., 2012; Khan et al., 2022; Ondrasek et al., 2022). The accumulation of salts in soil primarily results from natural salt deposits and irrigation with saline water, whereas human activities, including the discharge of untreated industrial effluents, overuse of agrochemicals, and unsustainable farming methods, can further exacerbate salinisation (Bartels and Sunkar, 2005; Chauhan et al., 2024). Soil salinity, characterised by electrical conductivity exceeding 4 dS/m, is a major abiotic stressor that impairs plant growth by disrupting its physiological and biochemical pathways (Kumar et al., 2022b). When salt deposits in soils, particularly sodium (Na^+^), chloride (Cl^-^), potassium (K^+^), and sulphate (SO_4_²^-^) (Balasubramaniam et al., 2023; Qadir et al., 2007; Stavi et al., 2021), It can interfere with the plant’s water absorption, induce ionic stress and ROS production. It further damages plant morphological, physiological and biochemical functions, resulting in impaired seed germination, stunted growth, chlorosis, photosynthesis inhibition, nutrient imbalance, oxidative stress and electrolyte leakage, ultimately leading to cell death (Balasubramaniam et al., 2023; Rafaliarivony et al., 2023) and inhibited plant growth and yield (Ahanger and Agarwal, 2017).

**Figure 1:**
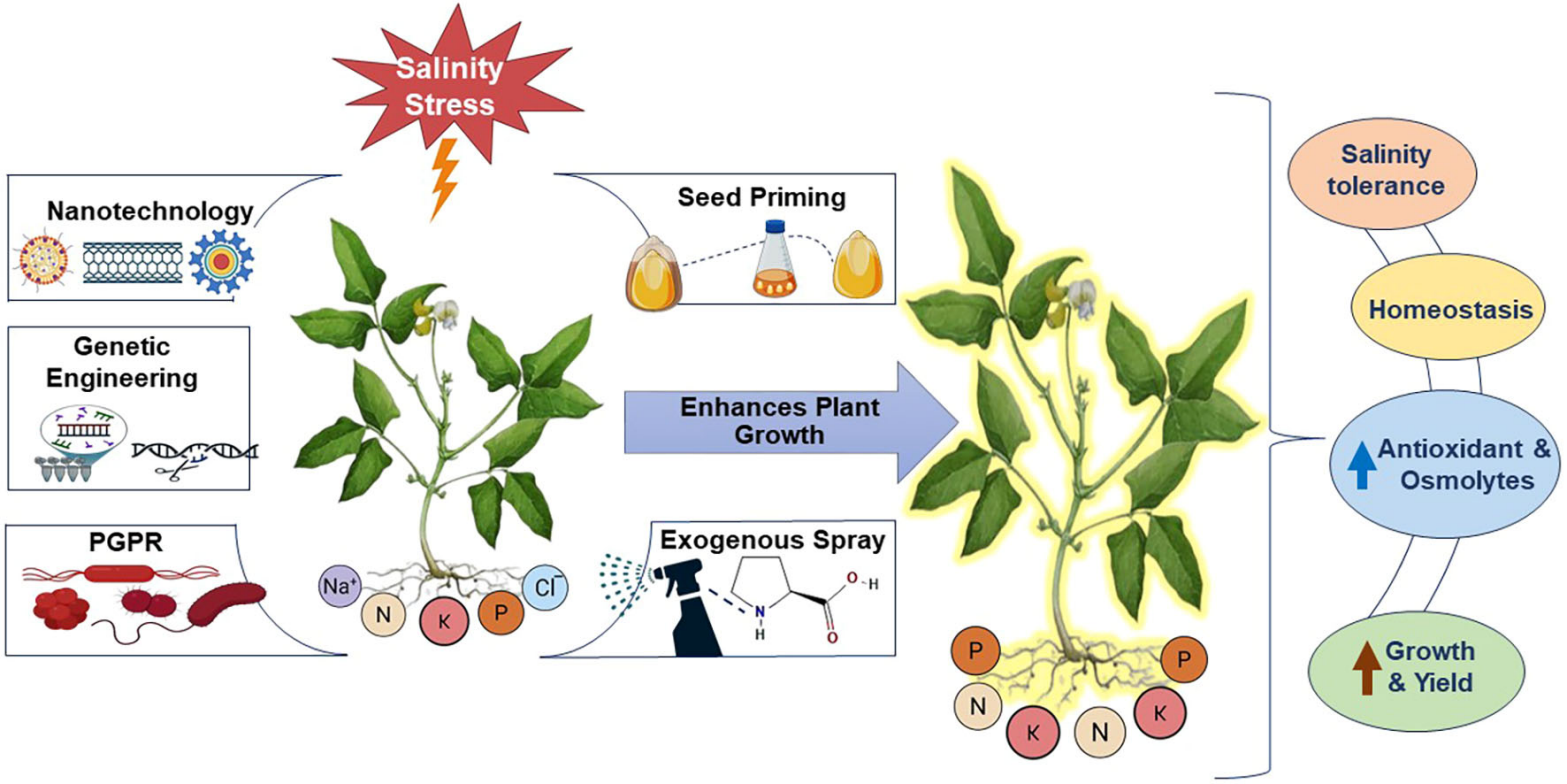
Emerging technologies to mitigate salinity stress.

Although several studies have reviewed various aspects of salt tolerance in leguminous crops (Bouzroud et al., 2023; El Moukhtari et al., 2024; Joshi et al., 2021; Kirova and Kocheva, 2021; Rane et al., 2021; Zheng et al., 2023), no one has specifically focused on *Vigna* species or comprehensively addressed recent developments in salinity stress mitigation in *Vigna*, which is the novelty aspect of this review compared to previous studies, which mainly focus on a broad spectrum of leguminous crops and general abiotic stress responses. This review presents a comprehensive and contemporary analysis of strategies to mitigate salinity stress in *Vigna* species, integrating insights from physiological, biochemical, and molecular perspectives. Additionally, **Figure 1** highlights new technologies and methods like nanotechnology, priming, exogenous compound application, plant growth-promoting rhizobacteria, and advanced biotechnological interventions as promising ways to improve salt tolerance and provide sustainable approaches for enhancing stress resilience and productivity in *Vigna* species.

## 2 Bibliometric analysis

**Figure 2:**
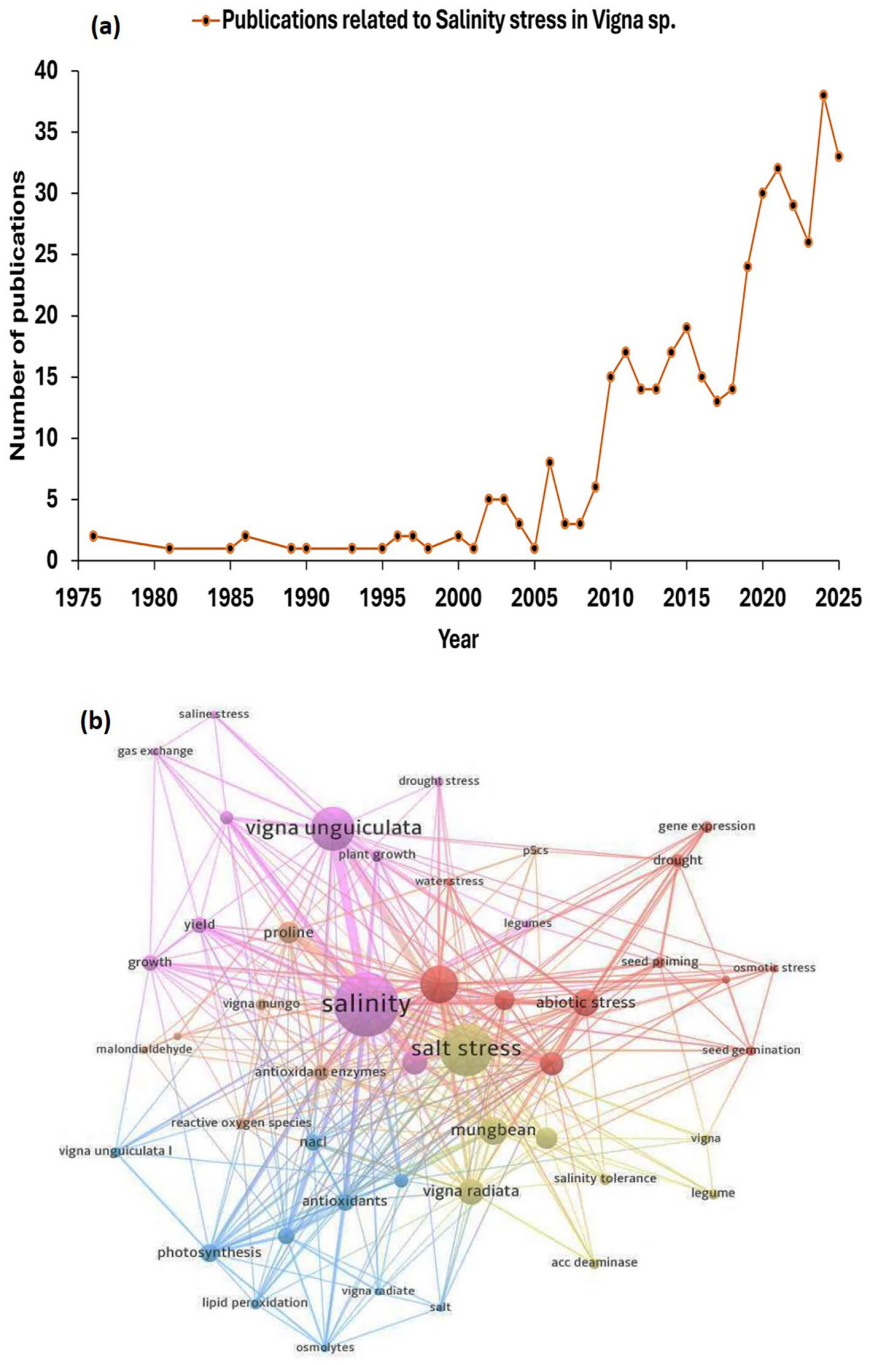
Bibliometric analysis on salinity stress in *Vigna* species, based on Scopus data (1976-2025). **(a)** Growing scientific publications on salinity stress in *Vigna* species over the years. **(b)** Major keywords related to salinity stress in *Vigna* species. The network visualisation was generated using VOSviewer^®^ software.

The scientific community’s interest in evaluating the impacts of salinity stress in *Vigna* species is constantly increasing, as evidenced by the increasing number of publications retrieved from the Scopus database, as shown in **Figure 2a**. The keywords “*Vigna*” and “salinity stress” showed 427 overall results, including book chapters, review papers, and original research. We screened bibliometric analysis for original research only, which provides 401 articles (1976–2025) using these keyword search. The resulting network visualisation of research trends in salinity stress in *Vigna* species, derived from Scopus data, highlights four major interconnected research areas as illustrated in **Figure 2b**. The top-right red cluster mainly focuses on abiotic stress related to seed germination, priming and seedling growth. The central pink cluster concentrates on leguminous plant growth, particularly in *Vigna unguiculata* under salinity stress. The bottom-left blue cluster focuses on the biochemical analysis, emphasising key terms like antioxidants, lipid peroxidation, osmolytes and oxidative stress, particularly in *Vigna radiata* and *Vigna unguiculata.* The bottom-right cluster features the salt tolerance in *Vigna radiata*. In addition, a comprehensive literature review was conducted using databases including Scopus, Google Scholar to collect information on botanical description, salinity stress, physiological and biochemical responses, salinity stress mitigation, and related research trends in *Vigna* species. Studies that were out of scope, lacked sufficient details, or did not meet the relevance criteria were excluded. The selection process focused on relevance and relatedness to ensure only the most significant and appropriate information was included. This multidisciplinary study highlights the extensive approach utilised by researchers to understand the resilience of *Vigna* species under salinity stress.

## 3 Effect of salt stress on plants

In plants, salt stress, initially affecting the root system, has two main effects on plant growth: (i). osmotic stress, which restricts the water availability, and (ii). Ionic stress occurs when toxic ions like Na^+^ and Cl^-^ accumulate in excess (Acosta-Motos et al., 2017). The prolonged exposure to ionic stress caused by salt stress results in necrosis, chlorosis, and early leaf senescence (Flowers and Colmer, 2008; Vajjiravel et al., 2024). This also results in nutritional deficiencies, such as deficits in calcium (Ca²^+^) and potassium (K^+^), which impair enzyme activity, membrane integrity, and nutrient transport (Vajjiravel et al., 2024; Zhang et al., 2023). Besides ionic and osmotic stress, salinity-induced oxidative stress causes an excess of reactive oxygen species (ROS) (Ragab et al., 2022), which break down essential biological molecules, including proteins, lipids, carbohydrates, and nucleic acids (AbdElgawad et al., 2016; Hasanuzzaman et al., 2021). The simultaneous effects of oxidative, ionic, and osmotic stress caused by saline factors can decrease plant physiological, biochemical, and metabolic processes (Chauhan et al., 2024; Patankar et al., 2024).

**Figure 3:**
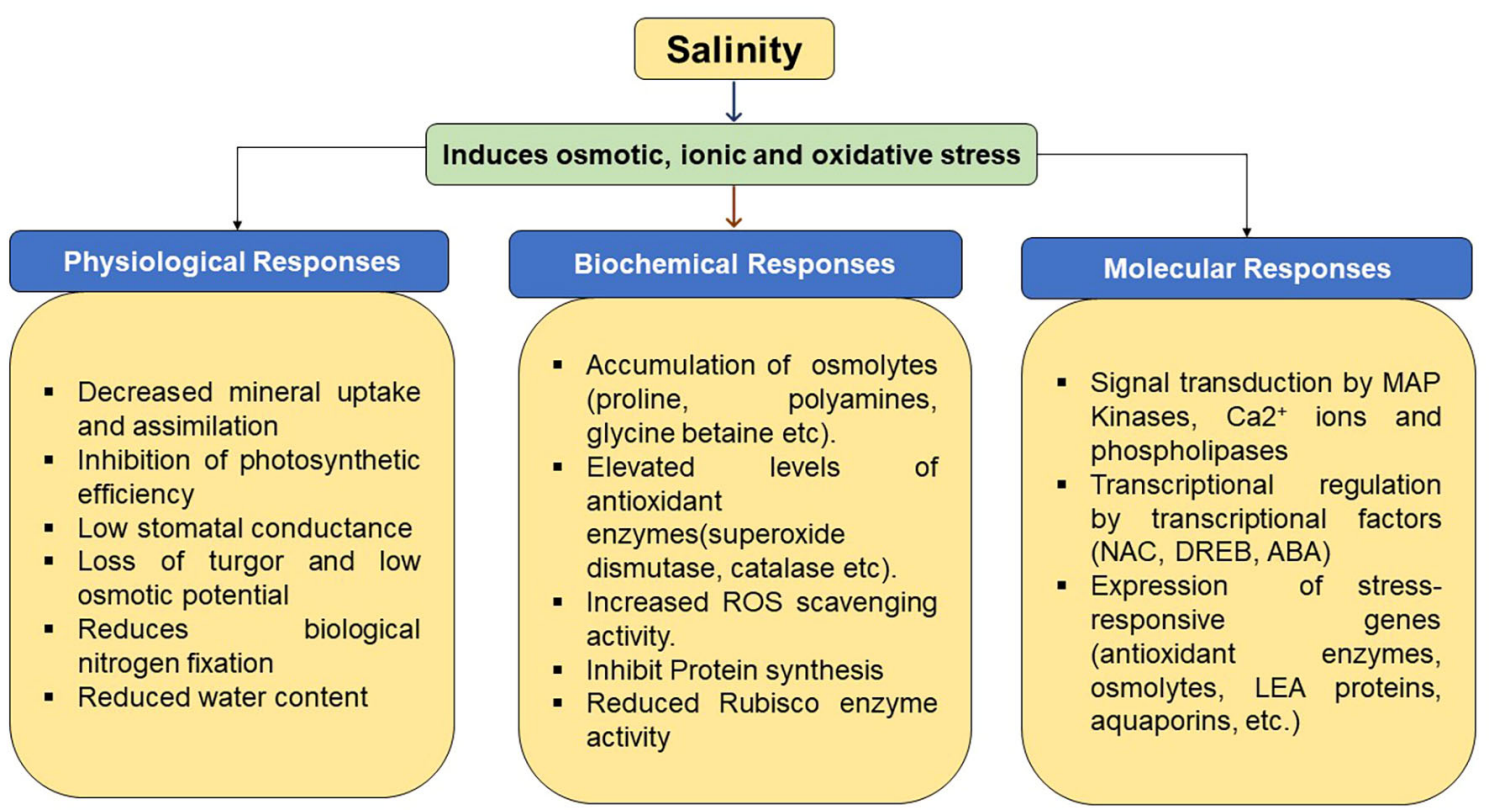
Physiological, biochemical, and molecular responses in plants to salinity stress.

Salinity stress severely affects legumes by reducing seed germination, plant growth, nodulation, and nitrogen fixation. It disrupts photosynthesis, carbon fixation, hormonal balance, and nutrient uptake, leading to delayed flowering, poor pod set, and decreased yield and quality (Farooq et al., 2017). The accumulation of excess salts in pulses induces anthocyanin pigmentation in leaves and stems, which in turn reduces germination and seedling establishment, highlighting their vulnerability to salt stress (Joshi et al., 2021; Kumar et al., 2016). In salt-sensitive legume species, osmotic and ionic stress, which are frequently associated with decreased hydrolytic enzyme activity and altered GA/ABA ratios, are one of the main ways by which salinity stress hinders seed germination (El Moukhtari et al., 2024). Moreover, it inhibits vegetative growth by lowering biomass, plant height, and leaf area (El Moukhtari et al., 2024; Lamsaadi et al., 2023). It also reduced tissue water potential in the plant physiological process, leading to restricting CO_2_ intake, photosynthetic efficiency and stomatal closure, finally resulting in growth inhibition of the plant (Dutta et al., 2018). In Molecular biology, salinity disrupts gene expression and may cause chromosomal abnormalities (El Moukhtari et al., 2024; Prajapati et al., 2023). Salinity stress disrupts several morphological, physiological, molecular, and metabolic functions in *Vigna* species, such as water intake, ionic balance and photosynthesis (Stavi et al., 2021). The three main forms of salt damage are oxidative, ionic, and osmotic stress, as illustrated in **Figure 3** (Dutta et al., 2018).

## 4 Adaptive mechanisms of plant tolerance under salinity stress

Plants mitigate salinity stress through physiological, biochemical, and molecular strategies, broadly classified into three main mechanisms: (i) ion exclusion, which prevents toxic accumulation of Na^+^ and Cl^-^ in roots; (ii) tissue tolerance, involving compartmentalisation of toxic ions within cells; and (iii) osmotic tolerance, enabling plants to maintain water balance under saline conditions (Jha et al., 2019; Liang et al., 2018; Munns and Tester, 2008; Negrão et al., 2017; Roy et al., 2014). The intensity of salinity tolerance in crops varies based on the plant genotype, the specific growth stage, and prevailing environmental conditions (Aghighi Shahverdi et al., 2018; Kumar et al., 2022b). Most glycophytes are generally salt-sensitive to NaCl, often showing reduced growth at concentrations between 50 and 100 mM, while halophytes can withstand salinity levels above 250 mM NaCl through specialised adaptive mechanisms (Flowers and Colmer, 2008; Vajjiravel et al., 2024).

Among *Vigna* species, currently, scientists have focused on *Vigna marina* (Burm.) Merrill, which grows on sandy coastlines (Chankaew et al., 2014), is a genetic resource for tolerance to salinity, and *Vigna luteola* (Jacq.) Bentham, which grows along riverbanks, is a genetic resource for tolerance to floods (Takahashi et al., 2016; Tomooka et al., 2014). Some of the *Vigna* species have previously been reported as salt-tolerant, as listed in **Table 1**.

**Table 1:** Salt tolerance genotypes or accessions of wild/domestic *Vigna* species.

S.No	Species (Wild/Domestic)	Genotype/Accession	(NaCl mM)	Gene(s)/QTLs/Trait	Effects	Ref
1	*V. nakashimae* (wild)	JP247291, JP107879	50–200	*POCO1*	No wilting up to 10 days; prevents Na^+^ accumulation in leaves; chlorophyll fluorescence is maintained even at 200 mM; biomass increased in salinised fields; maintains PSII efficiency through photosynthesis, but declines at 100 mM	(Iseki et al., 2016; Ito et al., 2024; Yoshida et al., 2016)
2	*V. riukiuensis* (wild)	JP108810	50–200	–	Survived >6–8 weeks in 200 mM; maintained photosynthesis at 100 mM; resilient to sudden transfer to 200 mM; produced 5× more biomass in saline fields; suppresses Na^+^ accumulation in whole plant body	(Iseki et al., 2016; Noda et al., 2022; Yoshida et al., 2016)
3	*V. luteola* (wild)	JP230741	100–400	–	Survives >4 weeks under 400 mM without visible damage; maintains transpiration and photosynthesis under 150 mM	(Naito and Wang, 2025; Yoshida et al., 2020)
4	*V. marina* (wild)	JP247202; subsp. oblonga	100–500	Saltol1.1, Saltol1.2 (QTLs at seedling and vegetative stages)	No salt damage at 500 mM for 4 weeks; enhanced transpiration and photosynthesis at 150 mM; altered root morphology to sustain water uptake at 200 mM	(Chankaew et al., 2014; Naito and Wang, 2025; Wang et al., 2024; Yoshida et al., 2020)
5	*V. trilobata, V. vexillata, V. luteola, V. marina* (wild panel)	JP205895, JP202334, JP233389, JP236246, JP235813	50–200	–	Accessions showed higher RQY and RSB values, extreme tolerance, and useful donors for cloning salt-tolerance genes	(Iseki et al., 2016)
6	*V. vexillata* (zombi pea, wild)	S261	250	QTLs qSaltol_3.1, qSaltol_7.1	Controls leaf wilt and plant survival under salt stress	(Laosatit et al., 2024)
7	Cowpea (*V. unguiculata*)	Acc. 211557; PI582422 and other accessions/varieties	0–250 mM; 6–12 dS/m	–	Tolerance at germination and seedling stage; tolerant accessions maintain higher biomass, germination %, and different salt-tolerant indices (GSTI, PHSI, RLSI, SFSI, RFSI, SDSI and RDSI); upregulation of stress genes in tolerant lines	(Afonso et al., 2025; Gogile et al., 2013; Islam et al., 2019; Ravelombola et al., 2017)
8	Mungbean (*V. radiata*)	112 accessions	100	*VrFRO8*	Upregulated under salt; regulates Fe²^+^/Fe³^+^ ratio; may lower SOD activity	(Liu et al., 2022)
9	Mungbean (*V. radiata*)	CO7 variety	0–125	Osmolyte pathways (proline, glycine betaine)	Increased carotenoids and osmolytes; maintained cell turgor and reduced water loss under 75–100 mM	(Mir et al., 2024)
10	Mungbean (*V. radiata*)	Wild accession CPI 100834; (GWAS panel)	50, 75	*Vradi07g01630* (AMT), *Vradi09g09510* (Glutaredoxin), *Vradi09g09600* (DnaJ)	Moderate tolerance (47–49% survival); Candidate genes for ion transport, redox regulation, protein folding under salinity	(Breria et al., 2020; Deeroum et al., 2024)

### 4.1 Physiological responses

Plants use a variety of physiological responses to promote growth and the stability of metabolic processes in response to salt stress. Key responses include the regulation of water status via relative water content, transpiration, and transpiration use efficiency, to maintain photosynthesis (Dutta et al., 2018). Under salt stress, plants regulate the Na^+^/H^+^ antiporter facilitates the transport of cytoplasmic Na^+^ into the vacuole for compartmentalisation (Dietz et al., 2001; Gupta and Huang, 2014). This mechanism is driven by two vacuolar H^+^ pumps: vacuolar pyrophosphatase (V-PPase) and vacuolar-type H^+^-ATPase (V-ATPase), with V-ATPase representing the predominant H^+^ pump in plant cells. V-ATPase activity is essential for plant survival under stress conditions to maintain solute equilibration, transportation and growth (Parihar et al., 2014). *NHX*s such as *NHX1* and *NHX2* regulate Na^+^ sequestration in the vacuole using Na^+^/H^+^ exchangers (Ismail and Horie, 2017). In *Vigna unguiculata* seedlings, V-ATPase activity increases in the hypocotyl under salt stress, whereas V-PPase activity is inhibited. This response shows with growing evidence that the Salt Overly Sensitive (*SOS1*) signalling pathway contributes to ion homeostasis and salt tolerance in cowpea (Otoch et al., 2001; Gupta and Huang, 2014). In response to salinity, plants minimise Na^+^ accumulation in their shoots to maintain ion balance. Na^+^ passes through root cells gradually through non-selective cation channels (NSCCs), which are classified as (VI-NSCCs), hyperpolarisation-activated (HA-NSCCs), and depolarisation-activated (DA-NSCCs). NSCCs allow Na^+^ and K^+^ to enter the roots, whereas calcium signalling controls these channels to prevent too much Na^+^ entry into the cells. Maintaining an adequate balance of Na^+^, K^+^, and Cl^-^ in the chloroplasts of salt-tolerant plants promotes effective photosynthesis and reduces the effects of salinity (Arif et al., 2020; Ismail and Horie, 2017).

### 4.2 Biochemical responses

Biochemical adaptations of salt tolerance involve the regulation of stress hormones (salicyclic acid, abscisic acid) (Verma et al., 2016), antioxidant enzymes (APX, POD, PPO, etc.), nonenzymatic antioxidants [ascorbic acid (vitamin C), phenolic acids (caffeic acid, ferulic acid), flavonoids (quercetin, kaempferol), alkaloids (polyamine, vinblastine, vinoline)] (Khan et al., 2025; Kumar et al., 2023; Shomali et al., 2022)and osmoprotectants, including amino acid (proline), quaternary ammonium compound (glycine betaine) (Mehta and Vyas, 2023) and carbohydrates/soluble sugars (glucose, fructose, trehalose) (Gupta and Huang, 2014), which are essential for retaining cellular homeostasis in areas of salt stress (Khalifa et al., 2024). *Vigna* species improve salt tolerance by accumulating osmoprotectants such as proline and glycine betaine, which maintain osmotic balance. Salt-tolerant mungbean cultivars, like ‘T44’, exhibit significant increases in these osmolytes in both roots and shoots at the seedling stage under NaCl stress (HanumanthaRao et al., 2016). This response is further enhanced by Ca²^+^, which promotes osmoprotection and stimulates osmolyte biosynthesis. In salt-tolerant *Vigna*, increased proline levels are associated with reduced proline oxidase activity and elevated production of key enzymes, including y-glutamyl kinase and P5CR, in both roots and shoots. These changes facilitate efficient osmolyte synthesis in salt-stressed mung bean seedlings (HanumanthaRao et al., 2016; Misra and Gupta, 2005). A study involving 17 mung bean genotypes under salinity stress was conducted in the seedling stage. It shows that seedlings of “PKU-AKM 12-28” and “PKU-AKM 8802” have enhanced biochemical characteristics, such as photosynthetic pigments, proteins, secondary metabolites, osmolytes, and antioxidants were increased, and lower MDA content in the germination stage, indicating that these cultivars are naturally salt-tolerant varieties possessing inherent biochemical adaptations to salinity stress (Mankar et al., 2021).

### 4.3 Molecular responses

Molecular responses to salinity stress in *Vigna* species include the expression of genes responsible for the osmolyte biosynthesis, which increases the osmotic potential of cells. Additionally, these genes encoding antioxidant enzymes contribute to the mitigation of oxidative stress (HanumanthaRao et al., 2016; Jiang et al., 2013). Calcium signalling pathways plays a vital role in resolving salt stress in *Vigna* species. Salt-induced increases in calcium activate specific calcineurin B-like proteins that interact with protein kinases (CIPKs) to regulate stress-responsive pathways. The isolation and characterisation of novel calcineurin B-like proteins highlight the role in calcium signalling pathways under salinity stress (HanumanthaRao et al., 2016; Imamura et al., 2008). Molecular responses induced by salinity stress include the expression of stress-responsive genes that encode transcription factors (*NAC, MYB, WRKY*) and ion transporters (*HKT, NHX, SOS1*). These genes control the processes involved in stress tolerance, cell division, and osmotic adjustment (Chakraborty et al., 2018). In mung beans, it has been demonstrated that overexpression of the vacuolar Na^+^/H^+^ antiporter gene *AtNHX1* increases salinity tolerance. Transgenic plants show better growth and development under salt stress, with less oxidative damage, and maintain improved ion balance. These findings imply that the overexpression of the *AtNHX1* gene may be a beneficial approach for developing salt-tolerant plants by assisting in the accumulation of sodium in vacuoles (Sahoo et al., 2016). Along with Na^+^/H^+^ antiporter genes, the salt overly sensitivity (*SOS1*) gene encodes a plasma membrane Na^+^/H^+^ antiporter that extrudes Na^+^ from cells (Zhu, 2003). Similarly, a study indicates that *V. marina*, one of the most salt-tolerant species in the genus, has higher expression of the *SOS1* gene. This increased *SOS1* activity enhances the plant’s ability to extrude sodium from the roots, subsequently contributing to its superior salt tolerance (Noda et al., 2025).

## 5 Microbial strategies for mitigating salinity stress

### 5.1 Plant-microbe interactions in salinity stress tolerance

Abiotic factors like salt drastically decrease crop yields, particularly in arid or semi-arid regions. Physical interventions, such as the application of gypsum to improve water management in salt-affected areas that are frequently utilised but tend to be expensive, ineffective, and have adverse impacts on the environment (Ali et al., 2023). These constraints emphasise the necessity for sustainable, biological strategies to mitigate salt stress.

Plant-microbe interaction is essential for increasing crop productivity and developing an innovative approach to minimise chemical fertilisers. The soil microorganisms (also known as plant-growth-promoting bacteria, or PGPB) that live or grow in the soil rhizosphere or within plant tissues, including roots (endophytes) (John et al., 2023; Meza et al., 2022), serve in a symbiotic relationship with plants. It improves soil nutrient availability, nutrient uptake and absorption, mineralises organic waste, sequesters salts and promotes plant growth. It allows plants to survive high concentrations of salt, temperature, and water stress and develops defence mechanisms against numerous biotic stresses of pests and diseases (Meza et al., 2022; Moreno Reséndez et al., 2018). Microorganisms in the rhizosphere region produce exogenous substances such as 1-Aminocyclopropane-1-carboxylate (ACC) deaminase, siderophores, indole acetic acid (IAA), and hydrogen cyanide (HCN) that improve nutrient uptake, suppress pathogens, and mitigate the impacts of salinity and sodicity (John et al., 2023). In particular, halotolerant PGPR or endophytes stimulate plant growth under salt stress by producing plant growth hormones, increasing disease resistance, enhancing mineral solubilisation and improving nitrogen fixation (Oviya et al., 2023). Microbes that can withstand salinity to certain extent, such as *Rhizobium* sp*s*, have demonstrated promise in increasing crop resistance to salinity stress and increasing soil fertility (Ali et al., 2023).

### 5.2 Role of ACC deaminase and siderophore production

High salt concentrations in plant cells induce oxidative damage and increase ethylene synthesis, because 1-aminocyclopropane-1-carboxylate (ACC) is excessively converted into ethylene (John et al., 2023). This ethylene negatively affects the plant’s root architecture. It further leads to an impact on nutrient uptake, root nodules formation in legumes, subsequently affecting physiological functions such as photosynthesis, respiration and nitrogen fixation and morphological parameters such as reduced seed germination, stunted plant growth and reduced agricultural yield (Gupta et al., 2023). In this regard, halotolerant PGPR produces ACC deaminase, an enzyme that breaks down ACC, a precursor of ethylene, into α-ketobutyrate and ammonia (Gupta et al., 2023; Kour et al., 2024). Therefore, using PGP microorganisms as a potent bioinoculants in agricultural practices to produce ACC deaminase inhibits ethylene synthesis. This approach is a sustainable, effective, economical, and environmentally friendly way to counteract the negative effects of salt stress (Kour et al., 2024).

For plants, iron is an essential micronutrient that is necessary for numerous metabolic processes, respiration, DNA synthesis, chlorophyll formation, and photosynthesis (Sultana et al., 2021). However, high salinity reduces Fe bioavailability due to its oxidation and decreased solubility, leading to chlorosis, nutrient imbalance, impaired plant growth, and low crop productivity (Ferreira et al., 2019). To address this, using PGPR that produces siderophores which play a key role in enhancing iron availability under saline conditions. Siderophores are small, low-molecular-weight, high-affinity and iron-chelating compounds secreted by bacteria and fungi and some plants under iron-limited conditions (Timofeeva et al., 2022). They are synthesised through non-ribosomal peptide synthetases or polyketide pathways and released into the soil, where they bind Fe³^+^ to form stable Fe³^+^ siderophore complexes. These complexes are recognised by specific membrane receptors such as TonB-dependent transporters in Gram-negative bacteria, and transported into microbial cells via ATP-binding cassette (ABC) transporters (Choi et al., 2024; Galdino et al., 2024). Once inside the cytoplasm, iron is released either through reduction to Fe²^+^ or via enzymatic modification of the siderophore, making it available for essential metabolic processes, including microbial survival and competitive growth (Kim et al., 2025). By enhancing iron solubility from organic complexes in saline soils, siderophore-producing PGPR improve iron bioavailability for plants, thereby enhancing chlorophyll synthesis, photosynthesis, and overall growth under salt stress (Cen et al., 2024).

### 5.3 Applications of salt-tolerant microbes in *Vigna* species

In *Vigna*, the use of salt-tolerant PGPR provides long-term solutions to improve salt tolerance while also assisting in soil reclamation in saline environments (Kumawat et al., 2022). Fifty halotolerant strains were isolated from Gujarat’s saline soils to perform halotolerant PGPR activity on *V. radiata*. Out of these 50 strains, *Bacillus subtilis* (BR5) and *B. megaterium* (BN7) demonstrated remarkable salt tolerance (up to 18% NaCl) and substantially enhanced germination and root and shoot length in *V. radiata* (Patel et al., 2015). Similarly, in another study, endophytic salt-tolerant bacteria such as *Serratia quinivorans* (TMB9), *Pseudomonas extremorientalis* (TMB6) and *Bradyrhizobium japonicum* (TMB7) were found to improve *V. radiata* growth and biomass by 45%–61% while enhancing antioxidants and stress tolerance (Zahra et al., 2023).

**Figure 4:**
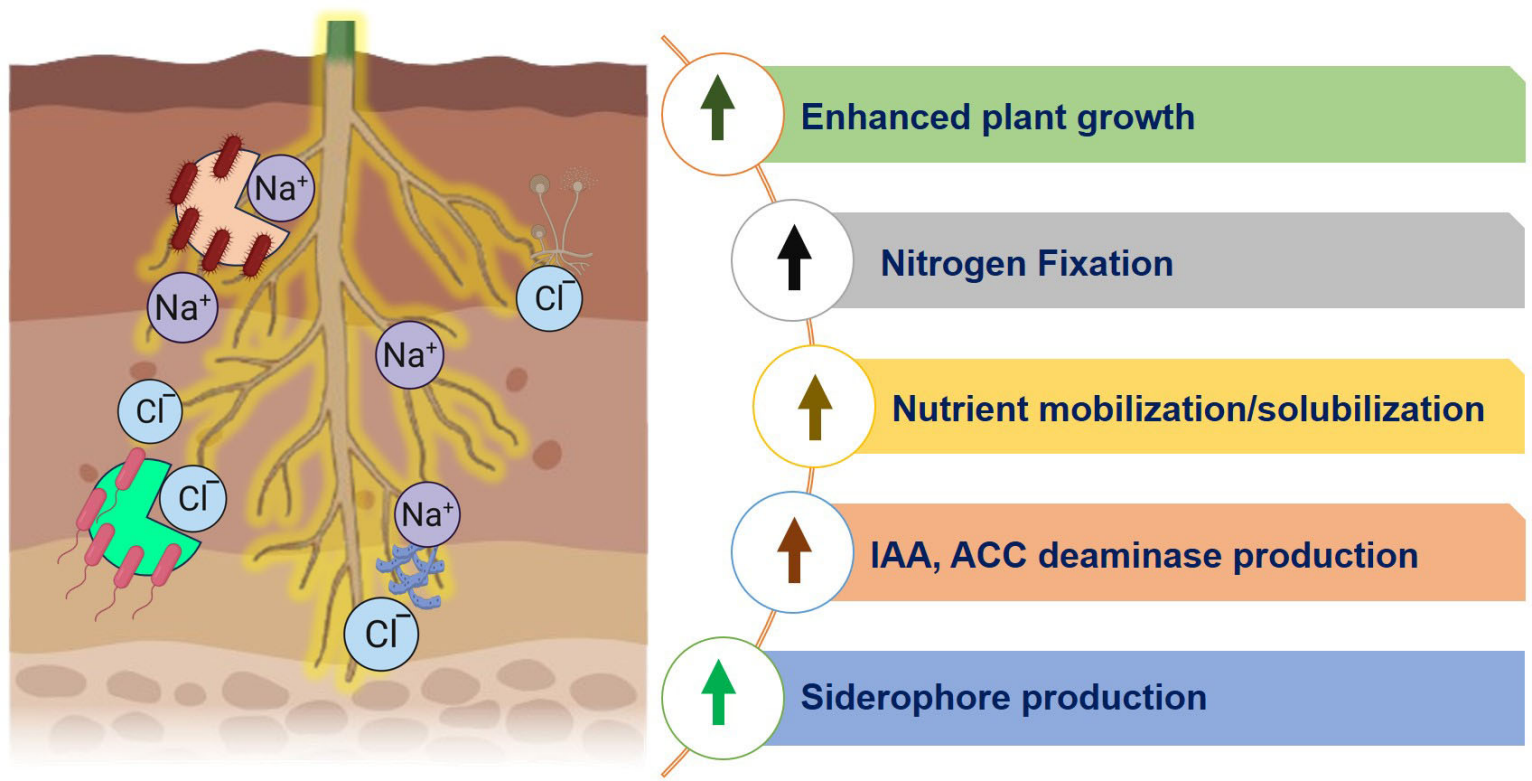
Microbial consortia to enhance plant growth.

Microbes mitigate salinity stress through several mechanisms. Salt-tolerant strains regulate sodium and potassium transport, aiding ion homeostasis. **Figure 4** shows that the microbial communities sequester salt ions to increase plant growth and yield. For example, *Bacillus cereus* Pb25, a halotolerant PGPR from saline-irrigated soil, when used as a bioinoculant, significantly boosted *V. radiata* growth under salt stress (9 dS/m) by improving plant biomass, nutrient uptake, yield, and antioxidant activity, while reducing sodium accumulation and oxidative damage. Additionally, it stimulated soil enzymatic and microbial activity (Islam et al., 2016). The halotolerant PGPR strain *Kosakonia sacchari* MSK1 exhibited exceptionally high salt tolerance, reportedly up to 1000 mM NaCl, a value unusually high for most PGPR. However, the strain produced IAA, ACC deaminase, and siderophores, and significantly enhanced *Vigna radiata* growth under salinity by improving biomass, chlorophyll content, and yield while reducing oxidative stress and ion toxicity (Shahid et al., 2021).

Salt stress has been demonstrated to drastically decrease root and shoot biomass in mung bean while concurrently raising oxidative stress indicators, including proline and glycine betaine. By improving ionic balance and mineral uptake, inoculating soil with *Rhizobium* strains significantly increases plant height, biomass, pod weight, and yield in mung beans (Ali et al., 2023). Furthermore, the salt-tolerant PGPR bacterial strains *Pantoea dispersa* and *Enterococcus faecium* significantly enhanced growth and yield under salinity stress, and they reduced Na^+^ accumulation and membrane damage while increasing antioxidants in *V. radiata* (Panwar et al., 2016). Additionally, salt-tolerant bacteria such as *Enterobacter* and *Cronobacter* species identified using 16S rRNA sequencing have been demonstrated in mung beans to maintain ion homeostasis by reducing Na^+^ accumulation and lowering ROS levels, while increasing proline, chlorophyll, and overall plant growth and development (Desai et al., 2023). When the plant grows in saline circumstances, the salt-tolerant CB 46 and cowpea cultivar 603 demonstrated enhanced growth and productivity with natural soil microbiota from samples SS-2 and SS-4, which included *Actinobacteria*, *Firmicutes*, and *Proteobacteria*, particularly *Bradyrhizobium* species. These treatments increased PGPR activity, suggesting that they may be able to mitigate the effects of salinity stress in cowpea farming (Mukhtar et al., 2020).

In addition, salinity stress induces ethylene, which inhibits root growth in mung bean. The application of ACC-deaminase-producing PGPR has proven effective in alleviating ethylene-induced stress in *V*. *radiata.* Beyond single-strain inoculation, integrated biofertilizer strategies involve the co-inoculation of multiple beneficial microbes to increase plant growth and yield. For Instance, Co-inoculation with *Rhizobium phaseoli* (M6 and M9) and PGPR (*Pseudomonas syringae* and *P. fluorescens*) enhanced growth, pod numbers, and total dry weight in mung bean under saline conditions (Ahmad et al., 2012). Similarly, A field-based biofertilizer application strategy involving co-inoculation with salt-tolerant microbes, including *Rhizobium* sp. LSMR-32, *Enterococcus mundtii* LSMRS-3, *Pseudomonas flourescens* LSMR-29 and *Enterococcus hirae* LSMRS-7, which significantly enhanced plant growth, germination, biomass, salinity tolerance, nodulation, nutrient uptake, antioxidant defence, and ion homeostasis, resulting in an 8.92% increase in spring mung bean yield (Kumawat et al., 2021, 2024). To effectively mitigate the salinity stress, an integrated approach by using the combination of L-tryptophan and *Rhizobium phaseoli* (strain N20) enhanced auxin production, nodulation, biomass and grain yield in *V. radiata* (Zahir, 2010).

Similarly, mung bean cultivars *“Inqelab mung”* exhibited maximum salinity tolerance when inoculated with salt-tolerant *Bacillus pseudomycoides*. Microbial diversity plays an essential role in agricultural productivity in stressed circumstances. Beneficial soil organisms regulate plant metabolic processes, assist nutrient uptake, and improve ion homeostasis, enabling plants to survive and grow in abiotic challenges such as salt, drought, and heat (Bilal et al., 2024). Likewise, inoculation with the endophytic fungus *Aspergillus awamori* has been shown to improve mung bean salt tolerance by increasing plant growth, nutrient uptake, and antioxidant enzyme activity (Ali et al., 2021). For legume crops such as *V. radiata, V. mungo*, and *V. unguiculata*, microbial treatments are long-term ways of minimising salt stress. Utilising salt-tolerant PGPR and endophytes can improve soil quality, increase production, and reduce the need for chemical fertilisers. Key microbial strains and their effects are summarised in **Table 2**.

**Table 2:** The impact of microbes on *Vigna* growth in saline conditions.

S. No.	Plant	Salt level (NaCl)	Microbial strain(s)	Effects	Ref.
1	*V. radiata*	*5% NaCl*	*Bacillus subtilis* (BR5), *B. megaterium* (BN7)	Strains enhance siderophore production, solubilise phosphate and produce IAA. It also increased the germination rate, shoot and root length	(Patel et al., 2015)
2	*V. radiata*	*-*	*Bradyrhizobium japonicum* (TMB7), *Pseudomonas extremorientalis* (TMB6), *and Serratia quinivorans* (TMB9)	Increased plant dry weight, increased proline, glycine betaine and proteins under salt stress	(Zahra et al., 2023)
3	*V. radiata*	1.41, 4 and 6 dS m^−1^	*Rhizobium isolates (Mg1, Mg2, and Mg3)*	Rhizobium strains, Mg3 inoculation improved plant growth, yield traits and nitrogen content at 6 dS m^-1^; Mg1 and Mg2 demonstrated excellent performance at 1.41 and 4 dS m-1 salinity levels	(Ali et al., 2023)
4	*V. radiata*	*-*	*Pseudomonas syringae*, *P. fluorescens, P. fluorescens Biotype G* with *Rhizobium phaseoli* (M6, M9)	Co-inoculation of microbes increased plant growth, yield traits, water use efficiency and relative water content	(Ahmad et al., 2012)
5	*V. radiata*	*-*	*Rhizobium phaseoli* (strain N20) + L-tryptophan	Increased plant height, nodule formation, biomass, and yield	(Zahir, 2010)
6	*V. radiata*	3 to 15 dS m^-1^	*Bacillus pseudomycoides*	The “*Inqelab mung*” cultivar with bacterial inoculum exhibits an increase in growth and yield characteristics, showing that *Bacillus pseudomycoides* inoculum mitigates salt stress	(Bilal et al., 2024)
7	*V. radiata*	–	Rhizosphere microbiomes	Enhanced plant growth with reduced stress markers to mitigate salt stress	(Anand et al., 2021; Dubey et al., 2022)
8	*V. radiata* (*spring mung bean*)	Salt-affected soils	*Rhizobium* sp. (LSMR-32) + *Enterococcus mundtii* (LSMRS-3)	Increase chlorophyll content, germination, and plant height. It also enhanced overall growth, yield, antioxidant and ion homeostasis	(Kumawat et al., 2021)
9	*V. radiata*	9 dS m^−1^	*Bacillus cereus* (Pb25)	Increased antioxidant activity; Decreased Na^+^ accumulation and oxidative damage. It also increased proline, potassium, nitrogen and phosphorus content	(Islam et al., 2016)
10	*V. unguiculata* (Cowpea 603, CB46)	Semiarid soils	Native soil microbiota (SS-2, SS-4)	Enhanced PGPR activity, growth and Development. It is a potent biofertilizer that supports plant development under salt stress and semiarid soils	(Mukhtar et al., 2020)
11	*V. radiata*	1000 mM	*Kosakonia sacchari* (MSK1)	Inoculation with MSK1 enhances biomass, chlorophyll and yield; It also produces IAA, ACC deaminase, and siderophore to promote plant growth	(Shahid et al., 2021)
12	*V. radiata*	5 and 10 dS m^−1^	*Pantoea dispersa and Enterococcus faecium*	A combination of PGPR shows decreased Na^+^ and membrane damage; Raised antioxidants, growth, and yield	(Panwar et al., 2016)
13	*V. radiata*	150 mM	*Aspergillus awamori* (endophyte)	Inoculation enhances seedling growth, biomass, chlorophyll, antioxidant enzymes, nutrient uptake and IAA levels	(Ali et al., 2021)
14	*V. radiata*	2 and 10% salt-affected soils	*Cronobacter, Enterobacter* spp.	Reduced ROS and Na^+^, increases growth and proline accumulation; It produces IAA and siderophore and also enhances root and shoot length	(Desai et al., 2023)
15	*V. radiata*	4 and 6 dS m^−1^	*Rhizobium and Pseudomonas* spp.	Combined application improved the osmotic stress tolerance and salt-tolerant index in mung bean seedlings under saline conditions	(Ahmad et al., 2013b)
16	*V. radiata*	(0, 20, 40, 60, 80 g L^−1^)	*Enterobacter cloacae strain* KBPD (AA-P11)	It increases shoot length, root length, fresh and dry weight of the plant and also enhances chlorophyll content	(Bhise et al., 2017)
17	*V. radiata*	Salt-affected areas (EC = 4.1-6.7 dS m^-1^),	*Rhizobium and Pseudomonas* spp.	Co-inoculation of *Rhizobium* and *Pseudomonas* was the most effective treatment under salinity stress. It maintains higher relative water content and CO_2_ utilisation, which improves photosynthesis, water-use efficiency, and chlorophyll levels. Additionally, it enhanced phosphorus and protein accumulation in mung beans in field conditions.	(Ahmad et al., 2013a)
18	*V. mungo*	2% NaCl	*Halotolerant rhizobacterial* spp.	A plant having a higher germination percentage, shoot length, photosynthetic rate, and chlorophyll content with the inoculant in salt stress	(John et al., 2023)
19	*V. radiata*	150, 200 and 250 mM	*Aspergillus niger* (VN2)	It enhanced root and shoot length, biomass, leaf number, Protein concentration, relative water content, and chlorophyll content in plants under salt stress	(Chauhan et al., 2025)
20	*V. radiata*	3.12, 5.46, 7.81 dS m^−1^.	*Enterobacter cloacae and Bacillus drentensis* + two Si levels (1and2kgha−1)	Increase growth, yield, salt tolerance index, stomatal conductance, chlorophyll and relative water content	(Mahmood et al., 2016)
21	*V. unguiculata* *(Baladi)*	(1.87, 3.16, 5.88, 9.12, 12.14) dS m^-1^	*single and dual inoculation with selected halo-tolerant isolates* (SARS-Rh3, SARS-Rh5) *of Bradyrhizobium spp*	In liquid media with 30 g/L NaCl, SARS-Rh5 showed the highest tolerance, and it increased plant height, dry weight of plant & nodules, and nitrogen content. In the Field trial, Dual inoculation (SARS-Rh3 + SARS-Rh5) increases nodulation, growth, and yield	(Omara and El-Gaafarey, 2018)

## 6 Nanotechnology to mitigate salinity stress

### 6.1 Role of nanotechnology in salinity stress alleviation

In recent decades, nanotechnology has become a rapidly growing field in agriculture, attaining prominence for its capacity to provide innovative and effective techniques that are crucial for sustainable farming (Zulfiqar and Ashraf, 2021). The potential application of nanoparticles (NPs) to improve agricultural production through abiotic stress mitigation, nutrient delivery, and other plant growth applications is being studied. NPs typically range in size from 10 to 100 nm and have a high surface area-to-mass ratio (Azameti and Imoro, 2023), which enhances reactivity and absorption. For example, various kinds of nanoparticles (NPs), including C, K, Ca, S, Ag, Cu, Fe, Zn, B, Si, and Ti, have been investigated in plant growth activity under both controlled and natural environments (Zulfiqar and Ashraf, 2021).

**Figure 5:**
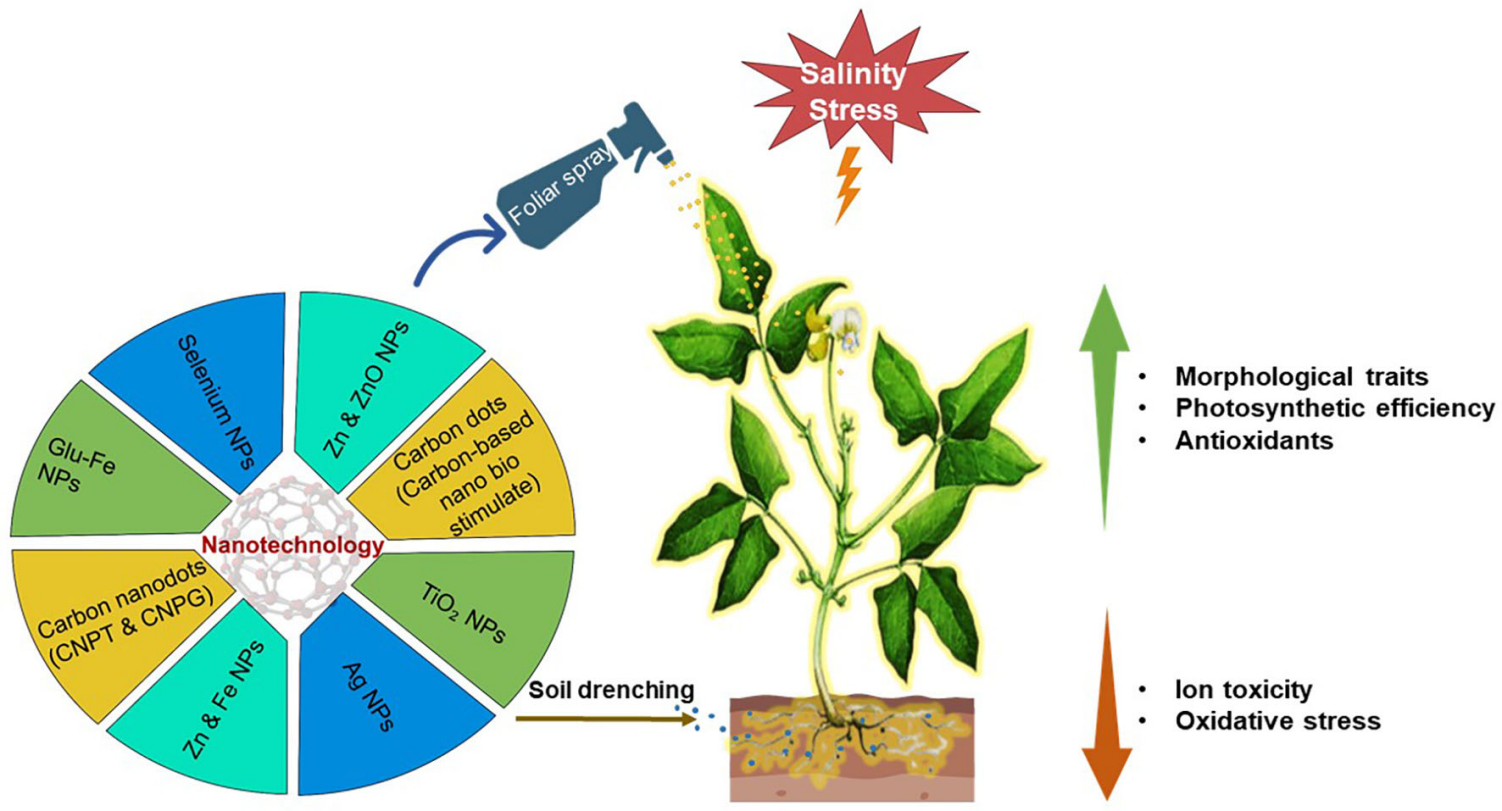
Nanotechnology responses in plants under saline conditions.

Furthermore, nanoparticles play a significant role in modern agriculture, providing precise and effective delivery of pesticides and herbicides and increasing soil physicochemical parameters, such as water-holding capacity (Rastogi et al., 2019). According to scientific research, nanoparticles can enhance plant growth to various abiotic stresses, specifically salt, by increasing physiological responses and overall productivity during growth (Ghassemi-Golezani and Abdoli, 2021; Zulfiqar and Ashraf, 2021). It can enhance seedling growth, plant development, stress resistance, and post-harvest quality. However, their beneficial effects are dependent on dose, as prolonged exposure can cause oxidative stress by producing excessive reactive oxygen species (ROS), which causes cellular damage (Muzammil et al., 2023). The effect of NPs is determined by their physicochemical properties, coating materials, stability, plant species, stage of development and environment (Muzammil et al., 2023; Turan et al., 2019). Despite these challenges, the specific action of NPs on plant cells via precision delivery, interaction, and improved metabolic processes provides an environmentally friendly substitute to conventional synthetic agrochemicals. They also improve photosynthesis, biomass and growth (Kim et al., 2018). The application of nanotechnology in agriculture, therefore, represents a cutting-edge, sustainable strategy for improving field crop production. In particular, to utilise innovative nano-remediation to alleviate salinity stress, which affects plant development, yield, and metabolic activities. Nanotechnology-based interventions provide promising tools for reducing salinity-induced damage, with unique nanomaterials showing an increase in antioxidants, ion management, and physiological enhancement in plants (Manzoor et al., 2024). **Figure 5** shows that nanotechnology enhances growth by providing macro and micro nutrients to the plant.

### 6.2 Application of nanotechnology in *Vigna* species under salinity stress

Glutamic acid-functionalized iron nanoparticles (Glu-Fe NPs) have been shown to enhance tolerance to osmotic stress in *V. radiata* by improving biomass, chlorophyll content and photosynthetic efficiency while increasing antioxidant activity (POD, SOD) and reducing the accumulation of oxidative stress markers (Ul Haq et al., 2023). An alternative nanotechnology-based strategy to improve salinity tolerance in *Vigna* species, especially in black gram, the exogenous treatment of selenium, which decreases sodium uptake, strengthens antioxidant defences, improves gas exchange, plant growth and PSII activity (Jawad Hassan et al., 2020). Similarly, exogenous zinc application under excessive salinity (150–200 mM NaCl) significantly reduces oxidative stress in *V. radiata* by improving the activity of antioxidant enzymes like ascorbate peroxidase (APX), catalase (CAT), and superoxide dismutase (SOD), which in turn enhances plant growth and salt tolerance (Al-Zahrani et al., 2021). In addition to zinc, using zinc oxide nanoparticles (ZnO NPs) externally has been shown to reduce salt stress by significantly improving growth indices such as shoot/root length, biomass, leaf area, and relative growth rate in *V. unguiculata*. These results demonstrate that ZnO NPs are an effective and sustainable method of mitigating salt stress (Mohammad Alabdallah and Saeed Alzahrani, 2020).

The application of nanotechnology to the reduction of salinity stress can be further developed by the synthesis of new nanomaterials. Sugar-terminated carbon nanodots (CNPT and CNPG), two novel nanomaterials, have demonstrated potential in lowering the levels of oxidative stress without causing toxicity. These nanodots promote seed germination, development, and stress tolerance while enhancing antioxidant defence mechanisms and maintaining ionic balance in mung beans (Sarkar et al., 2022). Furthermore, it has been shown that plant development is improved by carbon-based nanobiostimulants in salt environments. Application of these carbon-based nanobiostimulants enhances plant growth, yield, photosynthesis, and physiological performance of cowpea (*V. unguiculata*) at optimal concentrations of nano bio-stimulants (240–320 mg L^-^¹), particularly at higher salinity levels (4.0 dS m^-^¹). This suggests that the nano bio-stimulus may be able to mitigate growth reductions caused by salinity while improving water use efficiency (Oliveira et al., 2024).

Recent research has also looked into sustainable solutions for reducing salinity stress, including biosynthesised nanoparticles made from plant extracts. Under saltwater conditions, cowpea plants experience decreased growth, loss of photosynthetic pigment, and decreased seed protein. So, the foliar application of ZnNPs and FeNPs using *Psidium guajava* leaf extract alleviated these effects in cowpea, enhancing osmoprotectants, leaf pigments, protein content and seed yield. Moreover, ZnNPs improve the synthesis of proline, total protein and carbohydrates in both stressed and unstressed plants (Sheta et al., 2024). Likewise, green synthesised silver nanoparticles using *Pongamia pinnata* leaf extract in *V. mungo* have shown that it enhances 22% seed germination and 36% seedling growth, and further it increases root length, pod weight and grain weight, reducing ROS, promoting plant growth and acts as a plant growth enhancer (Krishnasamy et al., 2024). In *V. sinensis* (cv. *Pusa Komal*), a foliar spray of 50 ppm silver nanoparticles (AgNPs) significantly enhanced growth and root nodulation. The impact of Ag NPs on bacterial diversity, assessed via culture-based and DGGE methods, was notably altered, indicating concentration-dependent and species-specific microbial community shifts under nanoparticle exposure (Pallavi et al., 2016). In addition, the foliar application of biosynthesised TiO_2_ nanoparticles (10 mg/L) significantly enhanced mung bean growth by increasing root nodulation (67.5%), chlorophyll (46.4%), and soluble protein (94%). Further, application of TiO_2_ increases rhizosphere microbial populations and key soil enzyme activities, indicating TiO_2_ NPs’ potential as a plant growth-promoting nano nutrient (Raliya et al., 2015). Nanotechnology offers promising solutions for managing abiotic stresses like salinity in agriculture by enhancing plant resilience and resource efficiency. However, its sustainable use requires further research into its environmental impacts, dosage optimisation, and crop-specific effects. To increase sustainable agricultural production while reducing ecological threats, future initiatives should integrate nanotechnology with breeding, biotechnology, and precision farming.

## 7 Seed priming techniques: enhancing stress tolerance

### 7.1 Seed priming-induced stress resilience

**Figure 6:**
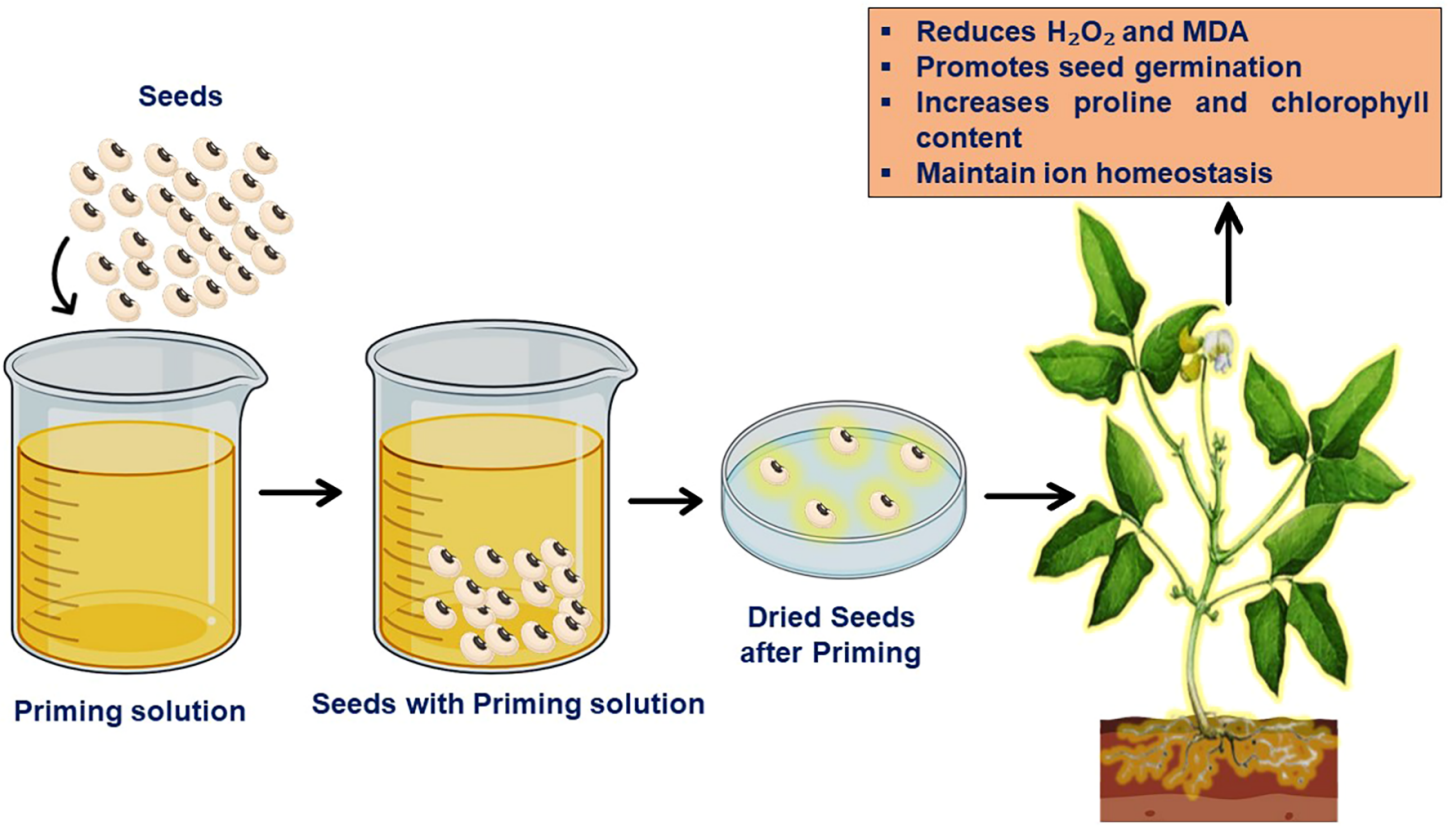
Seed priming, a pre-sowing method to increase plant growth.

In the plant life cycle, germination of seeds and seedling development act as critical phases that are especially susceptible to abiotic stressors such as drought, heat and salinity. Early growth phases in legumes suffer significantly under salinity stress, which reduces overall production and yield. A potential technique to improve seed performance under stress is seed priming, a pre-sowing treatment that involves controlled seed hydration and subsequent redrying. There are different methods of seed priming techniques to enhance seedling under salt stress, as demonstrated in **Table 3**. This process starts with physiological and biochemical changes before the germination stage, as shown in **Figure 6** (Nadeem et al., 2019). Seed priming prepares seeds to undergo radicle protrusion by initiating metabolic processes such as DNA replication, ATP production, protein synthesis, membrane repair and an increase in antioxidant enzymes. Together, these mechanisms improve stress resilience and promote seed vigour by promoting osmotic adjustments, improved membrane integrity, and decreased electrolyte leakage (Khan et al., 2022).

**Table 3:** Seed priming techniques to enhance salinity stress tolerance in *Vigna* species.

S.No	Plant species	Salt level (NaCl)	Priming type	Priming agent	Effects	Ref.
**1**	*V. radiata*	(0, 30, 60, 80, 90, 100, 200) mM	Hormonal Priming	Salicylic acid (0.5mM, 1 mM, 0.01%)	Enhances photosynthesis, nutrient uptake, biomass, and gas exchange	(Ashraf et al., 2023; Lotfi et al., 2020; Ogunsiji et al., 2023)
**2**	*V. radiata (Pusa Vishal/1431)*	150 mM		Salicylic acid and proline	Enhances germination, growth, and enzyme activity involved in metabolic pathways like the glyoxylate cycle, the pentose phosphate pathway, glycolysis and gluconeogenesis to enhance seedling growth	(Chauhan et al., 2023)
**3**	*Vigna radiata L*	5 dSm^-1^		proline	Enhances germination percentage, shoot & root length, shoot & root fresh weight and seed vigour index	(Sobahan, 2019)
4	*V. radiata*	100 mM	Chemical Priming	Sodium nitroprusside (SNP)	Reduces H_2_O_2_ and MDA, increases antioxidant activity, and improves growth and photosynthesis	(Roychoudhury et al., 2021)
5	*V. radiata*	30 and 50 mM		Thiourea	Enhances germination of seeds and shoot/root development under salt stress	(Jhanji and Dhingra, 2018)
6	*V. radiata*	(0, 25, 50, 75, 100, 125) mM		β-amino butyric acid (BABA)	Improved photosynthetic pigment levels, mitochondrial activities and Increased levels of proline, total protein, total carbohydrates, nitrate reductase activity, and antioxidant enzymes and It also decreased the amount of MDA content in the seedlings	(Jisha and Puthur, 2016)
7	*V. radiata (Pusa Vishal/1431)*	150 mM		KCl, KNO_3_, MgSO_4_	Improves germination, growth, and stress tolerance	(Chauhan et al., 2023)
8	*V. radiata (Pusa Vishal/1431)*	150 mM	Biopriming	Neem leaf extract, cow urine, moringa leaf extract	Promotes seed germination and reduces oxidative stress	(Chauhan et al., 2023)
9	*V. sinensis*	*10 and 25% seawater*		Seaweed liquid extracts (*Ulva fasciata*, *Laurencia obtusa*)	Improves enzymatic activity, biomass, and chlorophyll content	(Hussein et al., 2021)
10	*V. radiata*	100 and 200 mM		*Paecilomyces lilacinus* (KUCC-244) and *Trichoderma hamatum* (Th-16)	Increases growth, chlorophyll content and antioxidant enzymes. It further decreases oxidative stress markers (H_2_O_2_ and MDA)	(Irshad et al., 2023)
11	*Vigna radiata (IC- 119604, IC- 22456, IC- 282079, and IC- 38995)*	*-*		*Bacillus cereus*	These genotypes (IC- 119604, IC- 22456 and IC- 282079) have enhanced yield, growth, and salinity tolerance via rhizobacteria	(Jeeshitha et al., 2024)
12	*V. radiata*	0, 4 and 8 dSm^-1^	Nano priming	Nano-chitosan	Promotes seedling growth, antioxidant activity and salt stress tolerance	(Sen et al., 2020)
13	*V. radiata*	4.10 dSm^-1^ (saline soil)		Zinc oxide nanoparticles (ZnO-NPs)	Improves seed sprouting and antioxidant enzymes	(Sherpa et al., 2024)
14	*V. radiata*, *V. mungo*,	0–200 mM	Halo priming	CaCl_2_	Increase seed germination, growth, α-amylase activity, proline, and ion homeostasis	(Biswas et al., 2020; Mohanrao et al., 2023)
15	*V. unguiculata* (cowpea landraces (A18 and TZ2))	0, 85 and 170 mM		CaSO_4_/CaCl_2_	Promotes germination, shoot/root biomass, root, hypocotyl and epicotyl length in Algerian cowpea landraces	(Nabi et al., 2020)
16	*V. radiata*	100ppm and 200ppm	Nutri priming	ZnSO_4_	Enhance growth and development, increase proline content, and antioxidant activity	(Khan, 2020)
17	*V. radiata (variety- ‘Ramzan’)*	100 and 150 mM	Osmo priming	PEG	Increases dry mass, germination index, relative water content, proline, SOD, chlorophyll, and protein	(Ali et al., 2022)
18	*V. radiata (variety- ‘Ramzan’)*	100 and 150 mM	thermopriming	4 °C Low temperature	Enhances seed vigour, water use efficiency and antioxidant activity	(Ali et al., 2022)
19	*V. unguiculata*	10 dS m^-1^	Combined Priming	Biochar + osmopriming (CaCl_2)_	promotes biomass, chlorophyll, and oxidative stress tolerance to reduce Na^+^ toxicity in seedlings	(Farooq et al., 2020)

mM, millimolar; dS m^−1^, decisiemens per meter; ppm, parts per million; °C , degree Celsius.

### 7.2 Applications of seed priming in *Vigna* species

The application of chemical priming has shown great promise in increasing the salt tolerance of *Vigna* species, especially when combined with salicylic acid (SA) and sodium nitroprusside (SNP). Seed priming with salicylic acid (SA) at very low concentrations (e.g., 0.01% SA) has been reported to enhance biomass and nutrient accumulation in genotypes such as NM-2016 and NM-20-21 (Ashraf et al., 2023), whereas moderate concentrations (0.5Mm) (Ogunsiji et al., 2023). (0, 1.0, 1.5 mM SA) improve photosynthetic efficiency, gas exchange, nutrient uptake, antioxidant defence, and stress resistance in *V. radiata* (Lotfi et al., 2020). Other than salicylic acid, SNP priming increases the activity of antioxidant enzymes, growth and photosynthetic efficiency, which reduces oxidative damage, H_2_O_2_ and MDA levels under salt stress (Roychoudhury et al., 2021).

Biopriming *V. radiata* with organic substances such as cow urine, neem leaf extract (NLE) and moringa leaf extract (MLE) has demonstrated significant enhancements in seed germination and biochemical stress resistance under 150 mM NaCl stress (Chauhan et al., 2023). Similarly, the seaweed liquid extracts (SLEs) derived from *Ulva fasciata* and *Laurencia obtusa* priming on *V. sinensis* enhanced enzymatic activity, biomass, and chlorophyll content, revealing their effectiveness and ecologically harmless bio-stimulants for salinity tolerance (Hussein et al., 2021). Nanotechnology has been incorporated into priming techniques to enhance plant efficiency in saline environments. Nano-chitosan, a nano-priming agent, significantly increased growth, antioxidant activity, and stress tolerance in *V. radiata* grown in a saline environment (Sen et al., 2020). Zinc oxide nanoparticles at lower concentrations improve growth, germination, and antioxidant enzyme activity; but, at higher concentrations, they affect root growth and development (Sherpa et al., 2024).

Halo priming with mineral salts such as CaCl_2_ significantly increases the yard-long bean’s [*Vigna unguiculata ssp. sesquipedalis* (L.) Verdc] germination rate, seedling vigour, and yield performance. CaCl_2_ priming demonstrated the significantly higher germination index, branching, and pod yield among all other priming approaches, showing potential benefits in raising crop productivity and production (Karim et al., 2020). Similarly in *V. radiata*, seed priming with CaCl_2_ increased germination to 84% and α-amylase activity by 45%, showing that it can minimise salt stress and restore seed germination (Mohanrao et al., 2023). Halopriming has also been demonstrated to alter metabolic pathways and ion control, improving salt tolerance in *V. radiata* and *V. mungo* by triggering essential compounds like proline and aspartic acid (Biswas et al., 2020).

Other promising methods include nutri-priming with CaSO_4_ rapidly enhanced early seedling development and germination characteristics of the Algerian cowpea landraces (A18 and TZ2) [*Vigna unguiculata* (L.) Walp.] under varying salt conditions (0, 85 mM, and 170 mM of NaCl). Comparing CaSO_4_ priming agents to unprimed controls, the primed seeds showed improved root and shoot development and greater germination rates, suggesting increased salt tolerance (Nabi et al., 2020). In addition, nutri-priming with micronutrients such as ZnSO_4_ in *V. radiata* under salt stress enhances growth, proline content, and antioxidant activity (Khan et al., 2020). Furthermore, thiourea priming improved shoot-root balance and germination under salt and water stress (Jhanji and Dhingra, 2018). In cowpea, combining biochar with priming agents further enhanced plant growth, biomass, chlorophyll content, and oxidative stress resistance (Farooq et al., 2020).

## 8 Exogenous applications for mitigating salinity stress

### 8.1 Phytohormones in salinity stress alleviation

**Figure 7:**
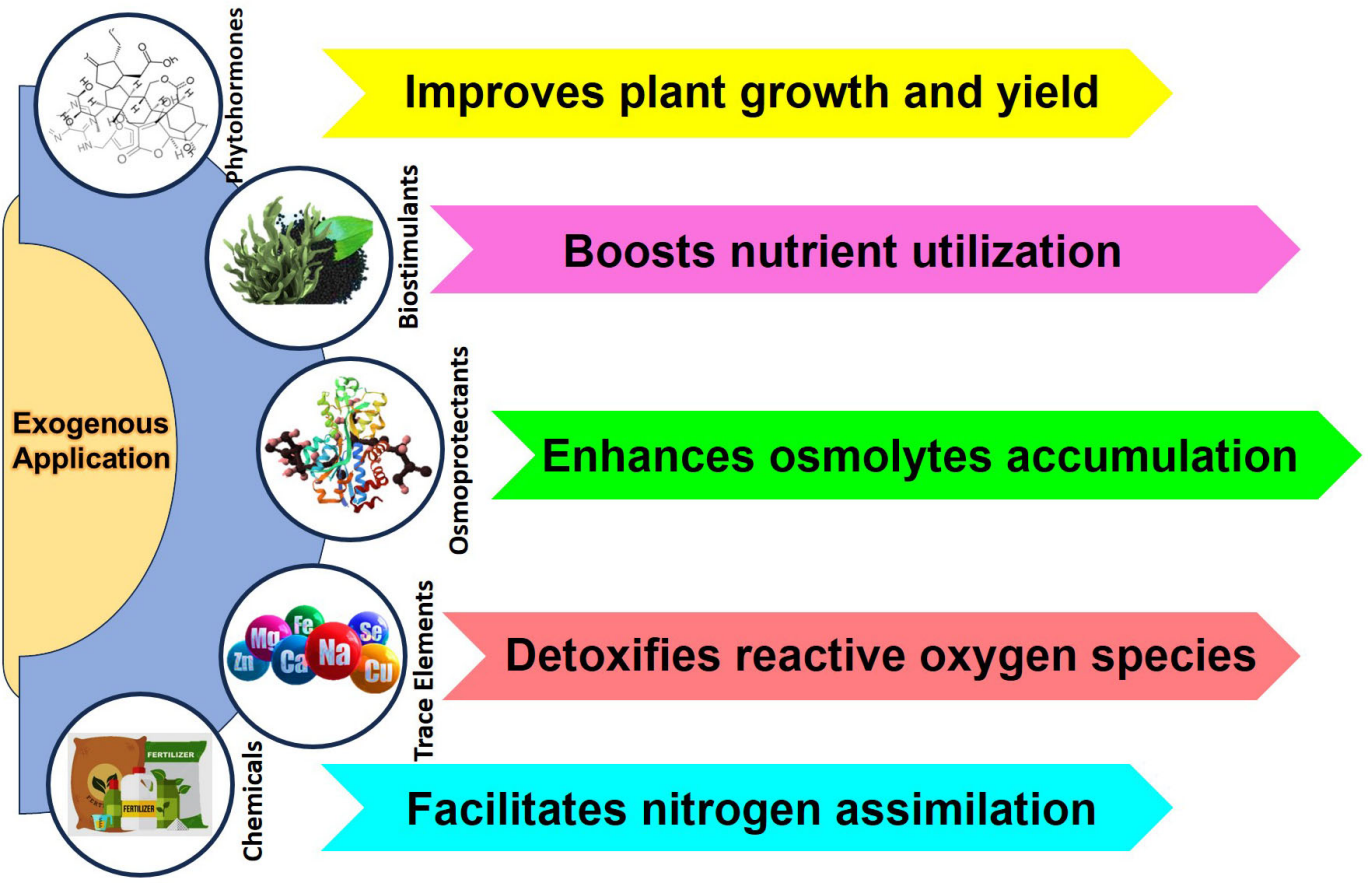
Exogenic treatment to maintain plant growth under salinity stress.

The production of legumes is severely restricted by salinity stress, which also affects physiological, biochemical, and yield-related characteristics. Many exogenous applications, such as phytohormones, antioxidants, osmoprotectants, and novel biostimulants, have shown significant promise in enhancing salinity tolerance in leguminous crops, especially in *Vigna* species, to mitigate the adverse effects of salinity, as shown in **Figure 7**. Exogenous plant tolerance to salt has been successfully increased by the use of phytohormones such as salicylic acid, abscisic acid, jasmonic acid, ethylene, auxins, gibberellins, cytokinins, strigolactones, and brassinosteroids, as well as nanotechnology-based treatments, which are crucial for regulating the growth and development of plants, as revealed in **Table 4** (Afeeza and Dilipan, 2024; Bilal et al., 2024; Wani et al., 2016). These phytohormones are crucial for signal transduction pathways, which allow plants to respond and regulate the environmental stressors such as salinity. This enhances crop productivity and overall stress resistance (Wani et al., 2016).

**Table 4:** Exogenous applications to mitigate salinity stress in *Vigna* species.

S.No.	Species	Category	Compound/ treatment	Concentration	Effects	Ref.
1	*V. radiata*, *V. mungo*, *V. unguiculata*	Phytohormones	Salicylic acid (SA)	(0.1, 0.5, 0.75 1.0) mM	↑Osmolytes, antioxidants, water potential, photosynthesis, nutrient uptake and growth, ↓Na^+^, Cl^-^, ROS, electrolyte leakage	(El-Taher et al., 2021; Rather et al., 2022; Syeed et al., 2021)
2	*V. radiata*		Ascorbic acid + SA	AsA (0, 50, 100, 150 mg L^−1^) and SA	↑Salinity tolerance, antioxidant activity ↓electrolyte leakage, oxidative stress markers	(Nawaz et al., 2021)
3	*V. angularis*		SA + Nitric oxide	SA (1 mM) + NO (100 µM)	↑photosynthesis, nutrient balance, osmolyte accumulation	(Ahanger et al., 2019)
4	*V. radiata*		SA + Sulphur	2.0 mM	↑Nitrogen assimilation, accumulation of osmolytes, and photosynthetic activity	(Hussain et al., 2021)
5	*V. radiata*		SA+ Si	–	↑Chlorophyll a, K^+^ Uptake, photosynthetic efficiency	(Ghassemi-Golezani and Lotfi, 2015)
6	*V. radiata*, *V. mungo, V. unguiculata*		Brassinosteroids (24-Epibrassinolide)	0.75–3 µM	↑plant growth, water, protein content, membrane stability, and chlorophyll,	(Kutay and Venkatesan, 2022; Singh et al., 2020; Sousa et al., 2022)
7	*V. radiata*	Polyamines	Spermidine, Spermine, Putrescine	1 mM	↑antioxidant, Glyoxalase activity, nutrient and water uptake	(Nahar et al., 2016)
8	*V. radiata*	Amino Acid Derivatives	γ-Aminobutyric acid (GABA)	1.5 mM	↑Growth, osmolyte, nitrogen metabolism, ↓Oxidative stress markers	(Ullah et al., 2023)
9	*V. radiata*	Biostimulants	Humic acid	0.3–0.5%	↑Yield, genotype-specific tolerance	(Patankar et al., 2024)
10	*V. sinensis*		Ascobin (Ascorbic + Citric Acid)	0.1–0.2%	↑Growth, Photosynthesis, yield, and nutrient uptake	(Abdelgawad, 2014)
11	*V. unguiculata*		Seed extracts of *Ammi visnaga* (ASE) and *Foeniculum vulgare* (FSE)	–	↑growth, yield, antioxidant activity, and nutrient uptake; ↓ oxidative damage and Na^+^ toxicity	(Desoky et al., 2020)
12	*V. mungo*		*Sargassum* sp.-derived seaweed liquid fertiliser (SLF)	10%	↑growth, chlorophyll, and relative water content, phytohormones, and antioxidants	(Afeeza and Dilipan, 2024)
13	*V. radiata*	Osmoprotectants	Proline, Glycine betaine	5–10 mM	↑GSH/GSSG content Antioxidants	(Hossain and Fujita, 2010)
14	*V. radiata*		Glutathione (GSH)	1–2 mM	↑Antioxidant, Gly I and Gly II, GSH/GSSG ratio; ROS detoxification	(Nahar et al., 2015)
15	*V. radiata*, *V. mungo*	Trace Elements	Sodium meta-silicate (Silicon)	1–2 mM	↑Na^+^/K^+^ balance, antioxidant enzymes, osmolyte accumulation, chlorophyll, and maintain stress-responsive genes	(Ahmad et al., 2019; Al Murad and Muneer, 2023; Kumar et al., 2024)
16	*V. radiata*		Selenium (Se)	1 ppm	↑Photosynthesis, antioxidant defence, pollen viability, and yield	(Kaur and Nayyar, 2015)
17	*V. radiata*		Se + Methyl Jasmonate	Se (1 ppm) + MJ (25 µM)	↑osmolyte, antioxidant activity, and ion homeostasis	(Alrashidi, 2022)
18	*V. radiata*		Zinc, Manganese, Glycine betaine	Zn-25 ppm, Mn-25 ppm, GB-50 ppm	↑Stress resilience, morphological and physiological traits, ↓ Oxidative stress markers (MDA), and electrolyte leakage	(Eisakhani et al., 2023)
19	*V. radiata*	Signaling Chemicals	Nitric Oxide	0.2mM	↑Germination of seeds, Chlorophyll accumulation, and stress tolerance	(Salahuddin et al., 2017)
20	*V. radiata*		Calcium (Ca)	10-15mM	↑Growth, vascular tissue integrity, and ↓ Chlorosis symptoms	(Khan et al., 2023)

### 8.2 Salicylic acid in salinity stress mitigation

Salicylic acid (SA), the most widely utilised exogenous phytohormone, reduces abiotic stressors such as salt stress. It is a naturally occurring phenolic phytohormone that enhances osmolyte (proline and glycine betaine) and maintains endogenous signalling pathways to induce tolerance to different biotic and abiotic stressors, and it also enhances enzymatic and non-enzymatic antioxidant properties in plants’ response to salinity stress (Janah et al., 2021; Kumar et al., 2022a; Rajeshwari and Bhuvaneshwari, 2017). Salicylic acid application improves plant water potential and antioxidant activity, and it reduces ion toxicity, oxidative damage, and lipid peroxidation by lowering Na^+^, Cl^-^, H_2_O_2_, TBARS (malondialdehyde), and electrolyte leakage in *V. radiata* (Khan et al., 2010). It also increases glycine betaine accumulation through increased methionine synthesis and suppresses ethylene production via ACS inhibition in *V. radiata* under NaCl stress (Khan et al., 2014). Additionally, salicylic acid elevates glutathione levels and improves growth and photosynthetic performance to reduce salinity stress and maintain crop productivity in *V. radiata* (Syeed et al., 2021). In *V. radiata*, foliar spray of salicylic acid improved growth, nitrogen metabolism, protein content, reductase activity, and the salt-tolerant cultivars (NM-98, NM-92) grew more rapidly than the sensitive cultivars (NM-54, NM-13-1) (Akhtar et al., 2013). Similarly, SA improved growth and yield under 75 mM NaCl stress in *V. mungo* (ADT-5) while decreasing MDA and electrolyte leakage and increasing proline, chlorophyll, carotenoids, and antioxidant enzymes (Rather et al., 2022). Another study assessed the impact of salicylic acid (SA) on mung beans. In this experiment, *V. radiata* cultivars NM-98 and NM-54 under 75 mM NaCl have induced ROS accumulation, impairing photosynthesis (Pandey and Lal, 2018), while exogenous application of SA significantly improved plant growth, antioxidant defence mechanisms to mitigate ROS by reducing salt stress impacts, while SA on *V. unguiculata* enhanced plant height, leaf, stem weights, photosynthetic pigments, nutrient uptake (N, P, K), and improved anatomical structures under salinity stress (El-Taher et al., 2021).

The combined application of SA and ascorbic acid to improve salt tolerance in mung bean, especially in the tolerant NM-92 cultivar, by reducing electrolyte leakage, lipoxygenase activities, oxidative stress markers and enhancing antioxidant activity under 80 mM NaCl concentrations (Nawaz et al., 2021). Similarly, the integration of salicylic acid and nitric oxide alleviated the salt stress (100 mM NaCl) in *V. angularis* by enhancing the growth, photosynthesis, osmolyte accumulation, and enzymatic and non-enzymatic antioxidant activities. This treatment improved nutrient homeostasis by increasing nitrogen, potassium, and calcium levels while reducing oxidative damage significantly (Ahanger et al., 2019). The synergistic effect of salicylic acid (SA) and sulphur (SO_4_²^-^) effectively mitigates salinity-induced stress in mung bean (*V. radiata*) by enhancing nitrogen assimilation (NR, NiR, GS), increasing proline and GSH accumulation, regulating antioxidant enzymes (SOD, APX, GR), and improving photosynthetic efficiency while reducing ROS, lipid peroxidation, and ion toxicity (Hussain et al., 2021). Besides, combined treatments of salicylic acid (SA) and silicon (Si) improved PSII activity in *V. radiata* under salinity stress (up to 9 dS/m) by enhancing the chlorophyll a fluorescence parameter, while reducing Na^+^ accumulation, increasing K^+^ uptake, enhancing photosynthetic efficiency and the plant’s physiological tolerance to salt stress (Ghassemi-Golezani and Lotfi, 2015). In addition, the exogenous applications of various compounds have exhibited significant promise in mitigating salinity stress in legumes by improving physiological, biochemical, and molecular defence mechanisms.

### 8.3 Applications of exogenous treatments in *Vigna* species

Exogenous applications, such as polyamines including putrescine, spermidine and spermine, notably mitigated 200 mM NaCl-induced stress in mung bean by enhancing antioxidant and glyoxalase activities, reducing oxidative and methylglyoxal toxicity and restoring nutrient homeostasis, chlorophyll and water content (Nahar et al., 2016). Similarly, γ-aminobutyric acid (GABA) improved growth, biomass, photosynthetic pigments, osmolytes, antioxidant activity and nitrogen metabolism while reducing Na^+^, H_2_O_2_, and MDA accumulation under 50–100 mM NaCl (Ullah et al., 2023). Brassinosteroids (24-epibrassinolide), applied at concentrations between 0.75 and 3 µM, improved plant growth, membrane integrity, chlorophyll content, protein content, antioxidant activity, relative water content, and stress tolerance in *V. radiata* (Kutay and Venkatesan, 2022)*, V. unguiculata* (Singh et al., 2020), and *V. mungo*, with dose-dependent responses (Sousa et al., 2022). Exogenous Nitric oxide promotes seed germination, seedling vigour, chlorophyll accumulation, and stress tolerance indices in salt-stressed *V. radiata* (Salahuddin et al., 2017). Humic acid application enhanced salt tolerance and yield performance, with genotypic differences observed among *V. radiata* cultivars: ‘ML-131’ showed a greater yield response while ‘Pusa Baisakhi’ exhibited higher salt tolerance (Patankar et al., 2024). Foliar spray of Proline and glycine betaine improved homeostasis under 300 mM NaCl by increasing GSH/GSSG content, maintaining the redox state, and enhancing antioxidant enzyme activities, reducing H_2_O_2_ and lipid peroxidation (Hossain and Fujita, 2010). Likewise, exogenous GSH enhanced antioxidant and glyoxalase pathways by increasing ascorbate and GSH content, improving the GSH/GSSG ratio, and stimulating the activities of key enzymes, including APX, MDHAR, DHAR, GR, CAT, GPX, GST, Gly I, and Gly II, ultimately reducing ROS and methylglyoxal toxicity (Nahar et al., 2015). Additionally, foliar application of Ascobin- a combination of ascorbic and citric acid (2:1), effectively alleviated salinity-induced reductions in growth, yield, photosynthetic pigments, and nutrient uptake (N, P, K) in *V. sinensis* (Abdelgawad, 2014). The exogenous application of silicon (Si), particularly as sodium meta-silicate, has proven effective in mitigating salt adverse effects by enhancing antioxidant enzyme activities, maintaining Na^+^/K^+^ homeostasis, and promoting osmolyte accumulation (Ahmad et al., 2019). Si supplementation improves chlorophyll fluorescence, gas exchange, and pigment synthesis while reducing lipid peroxidation and ROS accumulation. Moreover, it restores nitrate reductase activity and modulates the expression of stress-responsive and Si transporter genes, as confirmed by proteomic and transcriptomic studies. Collectively, these molecular, physiological and biochemical adjustments highlight the pivotal role of Si in enhancing mung bean resilience under saline conditions (Al Murad and Muneer, 2023; Kumar et al., 2024).

However, several exogenous nutrients and signalling molecules have demonstrated significant potential in mitigating salinity stress effects. Selenium (1 ppm) markedly improved plant growth, photosynthetic activity, antioxidant defence, and reproductive parameters such as pollen viability, while reducing Na^+^ accumulation and oxidative damage under 100 mM NaCl stress, by enhancing yield and salinity resistance (Kaur and Nayyar, 2015). The combined application of selenium and methyl jasmonate further amplified stress tolerance by promoting antioxidant activity, osmolyte accumulation, ion homeostasis, nitrate reductase activity, and membrane stability, while suppressing ROS production under saline conditions (Alrashidi, 2022). Similarly, foliar treatments with zinc, manganese, glycine betaine and calcium significantly improved morphological and physiological traits in mung bean and red bean under salt stress, including increased growth, chlorophyll, vascular tissue integrity, protein, and proline levels, and reduced oxidative markers such as MDA and Lipid peroxidation (Eisakhani et al., 2023; Khan et al., 2023).

Plant-derived bio-stimulants have shown great promise in improving legumes’ ability to withstand salinity by regulating their physiological and biochemical processes. In *V. unguiculata*, seed extracts of *Ammi visnaga* (ASE) and *Foeniculum vulgare* (FSE) enhanced growth, yield, antioxidant activity, and nutrient uptake under salt stress. However, ASE had been more effective in lowering ROS and Na^+^ toxicity, showing its advantage as a biological stimulant in a high salt stress environment (Desoky et al., 2020). Similarly, under saline conditions, *V. mungo* showed significant improvements in growth, chlorophyll content, relative water content, and important biochemical indicators following the application of a 10% seaweed liquid fertiliser (SLF) made from *Sargassum* species. Antioxidants and phytohormones were among the bioactive substances found in SLF’s LC-MS profile, suggesting its use as a long-term and efficient bio-stimulant for reducing salinity in legume production (Afeeza and Dilipan, 2024).

## 9 Biotechnological strategies for enhancing salt tolerance

### 9.1 Genetic and molecular approaches to enhance stress tolerance

In recent years, the development of stress-tolerant crops using innovative biotechnological techniques like synthetic biology, RNA interference (RNAi) technologies, and CRISPR/Cas9 gene editing has transformed genetic engineering by enabling precise, targeted modifications of plant genomes to improve tolerance to heat, cold, salinity, and drought (Mohapatra et al., 2024). Transcriptomic analyses of genetically modified plants show that *NAC* transcription factors control genes involved in food metabolism, photosynthesis, cell growth, and hormone signalling, improving plant growth and stress tolerance (El Moukhtari et al., 2024). In particular, salt tolerance in legumes has been enhanced through genetic modification of stress-related genes through genetic engineering/gene editing to reduce ionic stress (Nadeem et al., 2019). For instance, transgenic cowpea overexpressing *VuNAC1*, *VuNAC2* (NAC transcription factors) gene has shown improved photosynthesis, stress tolerance, nutrient availability, biomass and production (Srivastava et al., 2023). Other stress-responsive genes like *VrbZIP* and *VabZIP* from mung bean and adzuki bean have been identified via sequencing. These genes are involved in drought and salt stress tolerance mechanisms and may be potential targets for future molecular breeding and genetic engineering programmes (Wang et al., 2018).

### 9.2 Breeding and genome-wide association studies for salinity tolerance

Breeders have also been able to identify the key genes that contribute to salt tolerance traits by using QTL mapping and MAS to assist in developing salt-tolerant *Vigna* cultivars. Modern genomics techniques like WGS and NGS have improved the identification of DNA markers linked to salt tolerance. SNPs and SSRs, the most commonly utilised co-dominant markers in this MAS technique, enable targeted breeding, improving the effectiveness of producing crops resistant to salt stress (Farooq et al., 2017). Genetic diversity and salt tolerance genotypes have been identified using molecular markers, such as CBDP and SCoT. In this case, NaCl concentrations that varied from 0 to 200 mM caused significant changes in physiological, biochemical, and growth parameters in *V. radiata* (VBN3, VBN6, and CO8). These markers show that salinity stress-tolerant genotypes and genetic diversity in the species, which facilitates the identification of possible salt-tolerant genotypes for crop development (Dilipan and Nisha, 2024).

Genome-wide association studies (GWAS), a powerful method for identifying relevant alleles and markers for crop development, as well as for identifying genomic regions associated with complex quantitative traits. In contrast to conventional QTL mapping, GWAS uses historical linkage disequilibrium throughout the genome in a variety of natural populations to find relationships between markers and traits. In recent decades, genome-wide association studies have been widely applied to various *Vigna* species to identify genetic factors underlying complex traits, including salinity tolerance (Iqbal et al., 2025; Korte and Farlow, 2013). Recently, GWAS to evaluate salt tolerance in a cowpea multi-parent advanced generation inter-cross (MAGIC) population (234 lines) were evaluated and found significant variation in salinity-related characteristics. They discovered potential genes that encode calcium-dependent protein kinases, the salt tolerance gene *Vigun01g093100.1*, and Na+/Ca+ and K+-independent exchangers (Ravelombola et al., 2022). In a similar vein, the GWAS analysis using 5, 288 SNP markers identified QTLs linked to salt tolerance during the mung bean germination stage. Significant SNPs associated with salinity tolerance were found on chromosomes 7 and 9, including the genes *Vradi07g01630* (ammonium transport protein), *Vradi09g09600*, and *Vradi09g09510* (Breria et al., 2020; Sulthana Jafarullakhan et al., 2024).

### 9.3 Genetic engineering and gene-editing for salt stress tolerance

Genetic modifications across *Vigna* species have shown substantial improvements in salt tolerance by overexpressing significant stress-responsive genes. In black gram, the introduction of the Glyoxalase I gene from mustard, a member of the metallothionein superfamily, enhances salt tolerance by detoxifying the cytotoxic compound methylglyoxal into S-D-lactoylglutathione, thereby strengthening the plant’s oxidative stress response under saline conditions (Bhomkar et al., 2008). Similarly, in cowpea, overexpression of *VrNHX1* from mung bean increases salt tolerance by sequestering excess Na^+^ into vacuoles, maintaining ion homeostasis and lowering cytosolic toxicity (Mishra et al., 2014). Additionally, introducing the *P5CSF129A* gene from moth bean into pigeon pea considerably enhances proline production, which, in turn, minimises oxidative damage by scavenging free radicals, thereby enhancing salinity tolerance (Surekha et al., 2014).

In addition to transgenic techniques, the most recent technique was CRISPR-Cas9-mediated gene editing, which revolutionised the improvement of salt tolerance in *Vigna* species. Scientists use CRISPR/Cas9 to precisely edit or regulate genes related to salt tolerance in legumes. For instance, targeted mutagenesis of the *GmAITR* genes in soybean (*Glycine max*) improved the plant’s resistance to salt stress, demonstrating the potential of this approach for developing stress-tolerant legumes (Wang et al., 2021). In cowpea (*Vigna unguiculata*), CRISPR/Cas9-mediated knockout of the *SYMRK* gene efficiently disrupted nodule formation, demonstrating the gene’s essential role in symbiotic nitrogen fixation. This research constitutes the first successful application of genome editing in cowpea (*Vigna unguiculata*). This advancement is anticipated to significantly advance functional genomics studies of various agronomically important traits in Cowpea (Ji et al., 2019).

Furthermore, multi-omics techniques such as transcriptomics, proteomics, and metabolomics have considerably improved the molecular pathways underlying salt tolerance in legumes. Metabolomic studies have determined key osmolytes such as proline, glycine betaine, and sugars involved in osmotic adjustment, while transcriptomic analysis has revealed upregulation of genes related to antioxidant activity, stress-reduction biosynthetic pathways, and transcription factor regulations (Biswas et al., 2020; Chauhan et al., 2024).

## 10 Conclusion and future prospects

Salinity stress is a major challenge to the effective cultivation of *Vigna* species, influencing seed germination, seedling growth, nutrient uptake, photosynthesis, and overall yield and crop production due to ionic stress, oxidative damage, and osmotic stress. Although plant species possess inherent tolerance mechanisms such as osmolyte accumulation, ion homeostasis, antioxidant activity, and hormonal regulations, these mechanisms often become insufficient under conditions of saline irrigation or nutrient-poor soils. Recent advancements, which include external application of microbial inoculants (plant growth-promoting rhizobacteria), Osmoprotectants such as proline and glycine betaine, phytohormones & amino acids, nanotechnology, seed priming, and biotechnological techniques such as transgenics & CRISPR-Cas9 genome editing, have shown great promise in improving salinity tolerance. However, several research gaps still exist, such as restricted use of multi-omics techniques to analyse stress responses, field-level validation of nanomaterials and microbial consortia and a lack of reports on *Vigna* germplasm that can withstand salinity. Furthermore, future research should focus on integrated, crop-specific strategies that leverage the synergistic effects of microbiome engineering, nanotechnological and biotechnological approaches supported by bioinformatics and high-throughput phenotyping and genotyping. This review emphasises the significance of using lab findings in the field to support sustainable agriculture and global food production, and by integrating innovative and conventional approaches, including microbial engineering, genome editing and nano-biostimulants, to improve *Vigna* species tolerance to salinity stress.
